# Design, Docking Analysis, and Structure–Activity Relationship of Ferrocene-Modified Tyrosine Kinase Inhibitors: Insights into BCR-ABL Interactions

**DOI:** 10.3390/molecules30153101

**Published:** 2025-07-24

**Authors:** Irena Philipova, Mariyana Atanasova, Rositsa Mihaylova, Asine Dailova-Barzeva, Stefan M. Ivanov, Rumyana L. Simeonova, Georgi Stavrakov

**Affiliations:** 1 Institute of Organic Chemistry with Centre of Phytochemistry, Bulgarian Academy of Sciences, Acad. G. Bontchev Str. Bl. 9, 1113 Sofia, Bulgaria; irena.philipova@orgchm.bas.bg; 2 Faculty of Pharmacy, Medical University of Sofia, 1000 Sofia, Bulgaria; matanasova@pharmfac.mu-sofia.bg (M.A.); rmihaylova@pharmfac.mu-sofia.bg (R.M.); asinedailova61@gmail.com (A.D.-B.); sivanov@ddg-pharmfac.net (S.M.I.)

**Keywords:** tyrosine kinase inhibitors, imatinib, nilotinib, ferrocene, bcr-abl, leukemia, organometallic chemistry

## Abstract

Ferrocene (Fc), a redox-active organometallic scaffold, has attracted significant attention in medicinal chemistry due to its favorable physicochemical and pharmacological properties. The present study explores the therapeutic potential of novel Fc-functionalized analogues of imatinib and nilotinib, aimed at targeting BCR-ABL1+ chronic myeloid leukemia (CML) cells. A series of Fc-based derivatives (compounds **6**, **9**, **14**, and **18**) were synthesized by systematically substituting key pharmacophoric regions of the parent tyrosine kinase inhibitors with Fc units. The antiproliferative activity of these compounds was evaluated against four BCR-ABL1-positive leukemia cell lines (K-562, BV-173, AR-230, and LAMA-84), with imatinib serving as a reference drug. Biological assays revealed distinct structure–activity relationships. Compounds **6** and **9** demonstrated superior activity against the K-562 cell line, while compounds **14** and **18** exhibited enhanced potency and higher ligand efficiencies (LEs) against BV-173 and AR-230 cells compared to imatinib. Selectivity assays further indicated favorable toxicity profiles of compounds **9** and **14** toward malignant versus non-malignant cells. Molecular docking studies supported these findings, showing that Fc substitution alters binding interactions within the c-Abl kinase ATP-binding site while retaining key stabilizing contacts. Computationally predicted LEs showed strong correlation with experimental data, especially for K-562 and LAMA-84 cells, confirming the kinase as a relevant target.

## 1. Introduction

Ferrocene (Fc), a prototypical metallocene with a distinctive sandwich-like architecture, has emerged as a pivotal scaffold in the evolving field of bioorganometallic chemistry—a discipline formally conceptualized by Gérard Jaouen in the mid-1980s to describe the intersection of organometallic compounds and biological systems [1,2,3,4]. The integration of ferrocene into medicinal chemistry frameworks has garnered sustained interest due to its unique combination of physicochemical and pharmacological properties [5]. Among its notable features are high thermal and oxidative stability, low intrinsic toxicity, aqueous compatibility, and modular synthetic accessibility. These characteristics make Fc an attractive candidate for medicinal applications, particularly as a lipophilic, redox-active, and planar aromatic bioisostere of the phenyl ring [6,7].

**Figure 1:**
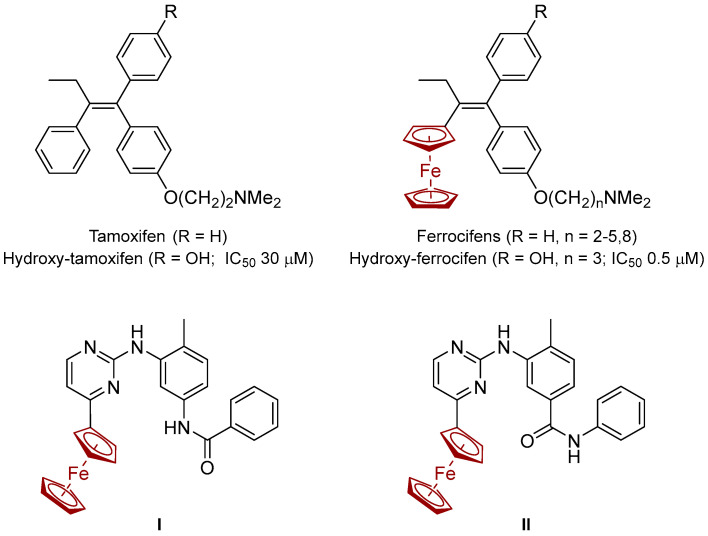
Structures and activities of hydroxi-tamoxifen and hydroxi-ferrocifen on hormone-independent (MDA-MB-231) breast cancer cells [10], and reported ferrocene-modified analogues of imatinib and nilotinib, (**I**,**II**) correspondingly [18].

The substitution of phenyl moieties with ferrocenyl groups in drug molecules often yields compounds with enhanced pharmacodynamic profiles. This strategy has been exemplified by the design of *ferrocifens*—Fc-modified analogues of tamoxifen—which have demonstrated broader and more potent anticancer activity than the parent compound (Figure 1) [8,9,10]. The therapeutic advantages of ferrocenyl-modified agents are attributed not only to improved membrane permeability and target binding, but also to the redox cycling capability of Fc under physiological conditions [11,12]. This redox activity leads to the generation of reactive oxygen species (ROS), which preferentially induce oxidative stress in cancer cells, exploiting their increased vulnerability to redox imbalance [13,14,15,16]. Such multi-target cytotoxicity may contribute to the circumvention of drug resistance, a hallmark challenge in modern oncology [8].

**Figure 2:**
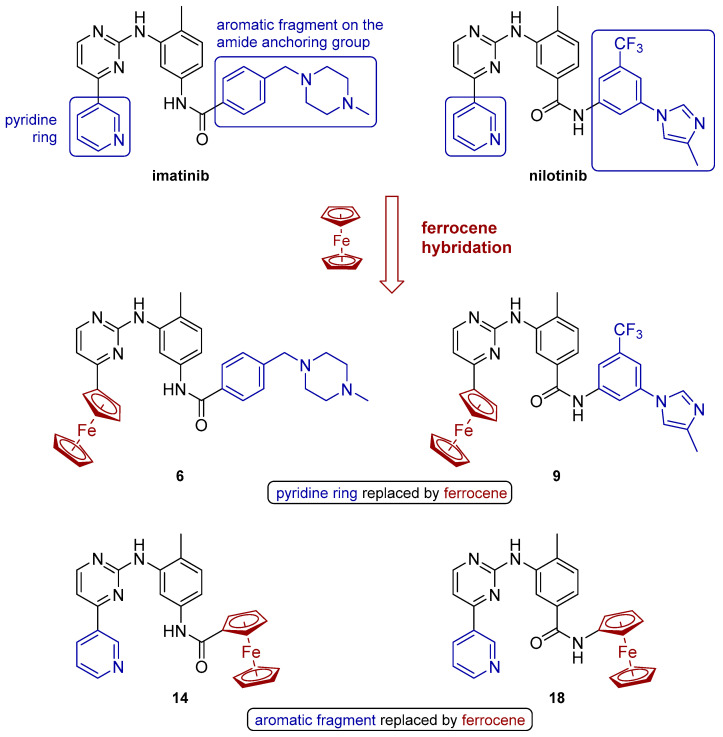
Design of the new Fc hybrids of imatinib (**6** and **14**) and nilotinib (**9** and **18**).

Building upon these principles, our previous work focused on the rational design of Fc-conjugated analogues of imatinib and nilotinib [17], the first-generation BCR-ABL tyrosine kinase inhibitors. Replacing the pyridyl fragment in the generalized structures of imatinib and nilotinib by a ferrocenyl unit (Figure 1), resulted in derivatives that retained or exceeded the anticancer efficacy of the parent drugs [18]. Encouraged by these findings, we expanded our efforts to the synthesis of full ferrocenyl analogues wherein Fc replaces both the pyridyl ring and auxiliary pharmacophoric moieties, including the amide anchor known to mediate key protein–ligand interactions. Thus, four ferrocene-modified analogues of imatinib and nilotinib were designed (Figure 2). Two of the structures, **6** and **9**, can be regarded as full analogues of the parent compounds, where the pyridine ring is replaced with a ferrocenyl moiety. In the other two hybrids, **14** and **18**, the metallocene is introduced into the serving as an anchoring group amide function. This structural evolution aims to integrate the redox and steric contributions of Fc into the molecular framework, potentially enhancing biological activity via dual mechanisms: kinase inhibition and ROS-mediated cytotoxicity.

The present study reports the synthesis, and preliminary biological evaluation of four novel Fc-functionalized analogues of imatinib and nilotinib. Emphasis is placed on structure–activity relationships and comparison with their non-ferrocenyl counterparts. These efforts contribute to the growing repertoire of bioorganometallic anticancer agents and underscore the therapeutic promise of ferrocenyl scaffolds in overcoming drug resistance and enhancing target selectivity.

## 2. Results and Discussion

### 2.1. Syntesis

**Scheme 1:**
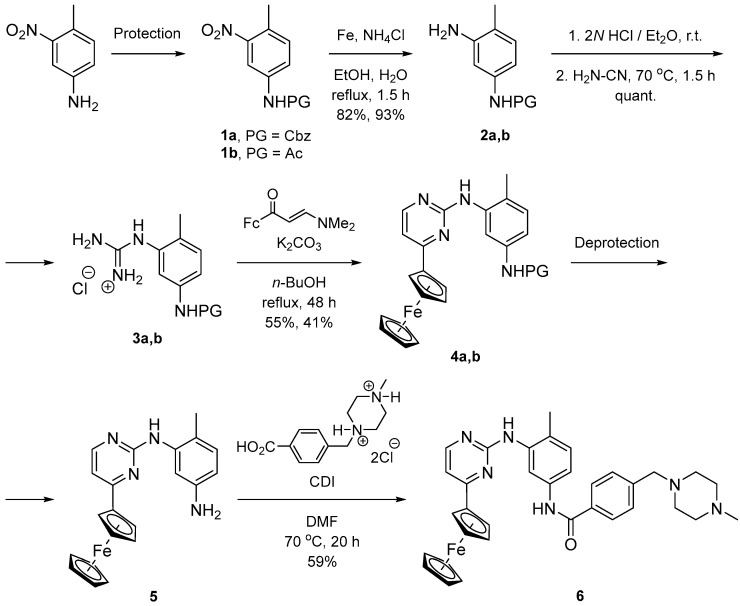
Synthesis of Fc analogue of imatinib. Protection: (a) CbzCl, NaHCO_3_, THF/H_2_O, r.t., quant; (b) Ac_2_O, NEt_3_, CH_2_Cl_2_, r.t., 3.5 h, 91%. Deprotection: (a) H_2_, Pd/C, MeOH, no reaction; (b) H_2_N-NH_2_.H_2_O, EtOH, reflux, 36%.

The synthetic strategy towards the full ferrocenyl analogue of imatinib was based on the differentiation of the two nitrogen functionalities by protection of the starting 4-methyl-3-nitroaniline (Scheme 1). The benzyloxycarbonyl was chosen as a classical amine protecting group. Thus, the reaction of the aniline with benzyl chloroformate quantitatively afforded the corresponding carbamate **1a**. A subsequent reduction with Fe/ammonium chloride in boiling ethanol/water converted the nitro compound to aniline **2a** in an 83% yield. The latter was transformed into its hydrochloride and melted with cyanamide to give the guanidinium salt **3a**. Cyclocondensation of (*E*)-3-(dimethylamino)-1-ferrocenyl-2-propen-1-one with **3a** constructed the key ferrocenyl-pyrimidine fragment, and afforded compound **4a** in a 55% yield. At this stage, deprotection and amide coupling were expected to give the target compound. Unfortunately, all our efforts to remove the carbamate amine protection failed, despite the use of different sources of Pd-catalysts and reaction conditions. This forced us to change the protecting group with the less elegant acetamide. Thus, acylation of the starting nitroaniline gave **1b** (91%), which was reduced to **2b** (93%) and quantitatively converted to its guanidine salt **3b**. Cyclization of the latter with the ferrocenyl-enaminone afforded the ferrocenyl-pyrimidine compound **4b** in 41% yield. Deprotection was achieved by hydrolysis of the acetamide with hydrazine hydrate in boiling ethanol to give aniline **5** in a 36% yield. Subsequent amide coupling of **5** with 4-[(4-methylpiperazin-1-yl)methyl]benzoic acid dihydrochloride and 1,1′-carbonyldiimidazole (CDI) as an activating agent furnished the desired compound **6** in a moderate yield.

**Scheme 2:**
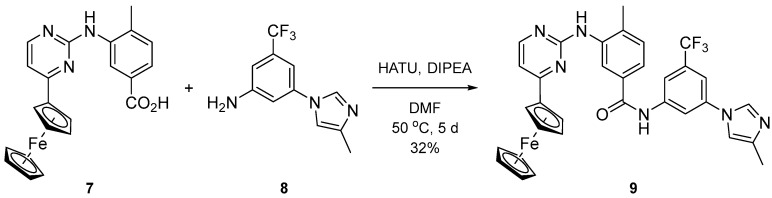
Synthesis of Fc analogue of nilotinib.

The synthesis of the full ferrocenyl analogue of nilotinib was based on the amide coupling of two key fragments. Carboxylic acid **7** was constructed starting from methyl 4-methyl-3-nitrobenzoate by Fe/NH_4_Cl reduction, guanidinium salt formation, cyclocondensation with ferrocenyl-enaminone, and basic hydrolysis of the ester [18]. Aniline **8** was synthesized via copper-catalyzed *N*-arylation of 4-methylimidazole with 3-bromo-5-trifluoromethylaniline in the presence of CuI/8-hydroxyquinoline [19]. The amide formation was successful when applying hexafluorophosphate azabenzotriazole tetramethyl uronium (HATU) as a coupling agent along with *N*,*N*-diisopropylethylamine (DIPEA) in DMF (Scheme 2).

**Scheme 3:**
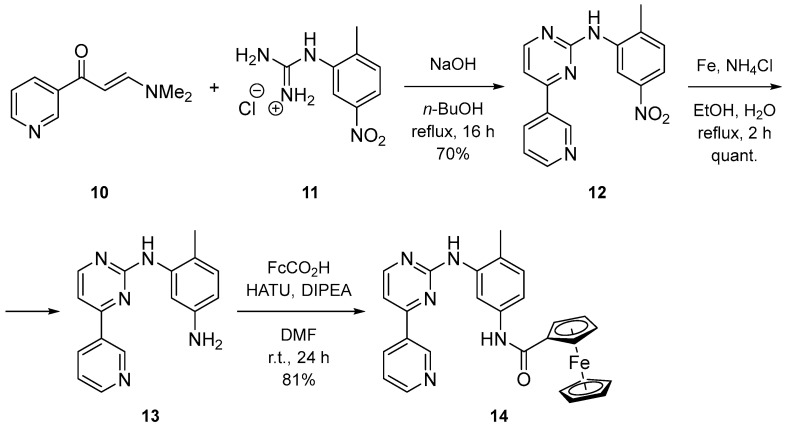
Synthesis of imatinib analogue with Fc anchoring group.

The imatinib analogue **14** with ferrocenecarboxamide as the anchoring group was synthesized by coupling aniline **13** and ferrocenecarboxylic acid in an excellent yield using HATU as the activating agent (Scheme 3). Amine **13** was prepared by cyclization of pyridinyl-enaminone **10** with nitro-guanidinium salt **11** to intermediate **12**, which was reduced by implementing the Fe/NH_4_Cl protocol.

**Scheme 4:**
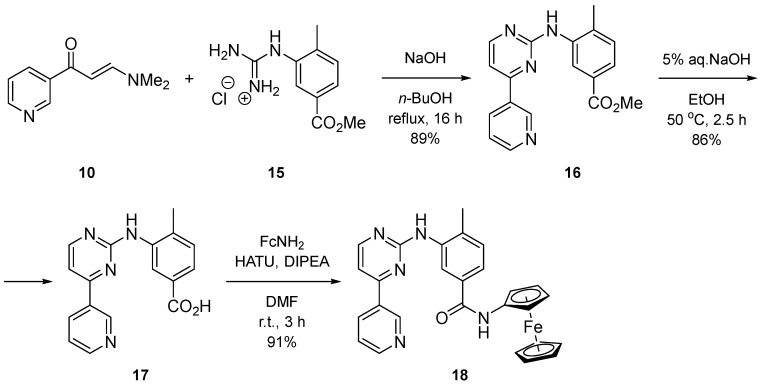
Synthesis of nilotinib analogue with Fc anchoring group.

Analogously, the nilotinib analogue **18**, which has an *N*-ferrocenyl amide as the anchoring group, was prepared from the carboxylic acid **17** and ferroceneamine (Scheme 4). The acid synthesis involves cyclization of enaminone **10** with the ester-guanidinium salt **15** to yield intermediate **16**, followed by its subsequent hydrolysis [18].

The purity of all tested compounds was evaluated by NMR and HRMS (Supplementary Materials).

### 2.2. Results from In Vitro Cytotoxicity Studies

A comprehensive analysis of the antiproliferative activity of the four ferrocene-modified tyrosine kinase inhibitors—compounds **6**, **9**, **14**, and **18**—reveals distinct structure–activity relationships across the panel of BCR-ABL^+^ chronic myeloid leukemia cell lines (BV-173, K-562, AR-230, and LAMA-84), when compared to the reference drug imatinib (Table 1).

Among the tested compounds, compound **6**, a ferrocene analogue of imatinib in which the pyridine ring is substituted with a ferrocenyl unit, demonstrates consistent moderate activity across all four cell lines. Notably, its potency surpasses that of imatinib against K-562 (IC_50_ = 29.9 µM vs. 45.5 µM for imatinib) and is nearly equivalent on the BV-173 line (33.6 µM vs. 22.8 µM). On AR-230, compound **6** exhibits a strong inhibitory effect (IC_50_ = 5.9 µM), comparable to imatinib (4.7 µM), confirming that the ferrocene substitution retains effective interaction within the kinase binding site. Compound **6** also shows moderate inhibition of LAMA-84 (25.4 µM), a cell line otherwise highly sensitive to imatinib (0.5 µM), although with significantly reduced efficacy.

Compound **9**, a structurally modified nilotinib analogue bearing a ferrocenyl group in place of its pyridine moiety, displays enhanced potency on K-562 cells (IC_50_ = 25.9 µM), demonstrating improved efficacy in that cellular system compared to imatinib. Its activity on BV-173 mirrors that of compound **6** (33.6 µM), but it performs poorly against AR-230 (143.8 µM), in contrast to the otherwise strong response observed in this cell line for other analogues. On LAMA-84, compound **9** again matches the moderate efficacy of compound **6** (25.4 µM), though it remains significantly less active than imatinib.

Compounds **14** and **18**, analogues of imatinib and nilotinib, respectively, in which structural modifications were introduced by modifying the serving as an anchoring group amide function with a ferrocenyl moiety, show an interesting activity profile. Both compounds are inactive against K-562 (IC_50_ > 200 µM), suggesting a loss of favorable interactions within the kinase domain in that context. However, their activity on BV-173 is superior to that of imatinib, with compound **18** being the most potent of all tested ferrocene derivatives on this cell line (IC_50_ = 15.1 µM), followed closely by compound **14** (17.9 µM). Their potency on AR-230 (5.1 µM for both) is also comparable to imatinib, confirming that despite their structural divergence, these analogues maintain productive binding within the ATP pocket in this cellular context. As with other compounds, activity on LAMA-84 is markedly diminished (26.8 µM for compound **18** and 27.3 µM for compound **14**), further highlighting the unique hypersensitivity of this cell line to imatinib.

It is particularly noteworthy that the AR-230 cell line displays the greatest overall sensitivity to all active compounds tested. Three of the four ferrocene derivatives (compounds **6**, **14**, and **18**) show IC_50_ values between 5.1 and 5.9 µM, close to the 4.7 µM IC_50_ of imatinib, indicating that AR-230 cells are broadly permissive to both traditional and structurally altered kinase inhibitors. This suggests either a kinase conformation highly compatible with modified inhibitors or a cellular context that allows efficient uptake and engagement. In contrast, LAMA-84 consistently exhibits a more pronounced responsiveness to imatinib, as its IC_50_ (0.5 µM) is drastically lower than that of any ferrocene analogue, pointing to a possibly stricter structural requirement or higher dependency on the canonical binding interactions lost in the modified analogues.

Selectivity toward malignant cells was also noted. Compounds **9** and **14**, in particular, exhibited favorable selectivity profiles, showing cytotoxicity at lower concentrations in leukemia cells relative to non-malignant murine fibroblasts (CCL-1). This suggests potential for therapeutic windows and safety advantages over non-targeted chemotherapeutics.

Together, these findings suggest that while ferrocene substitution can preserve or even enhance activity against certain leukemia models—particularly BV-173 and AR-230—the effectiveness is highly dependent on the structural context of the analogue and the specific cellular model. The divergence in activity profiles highlights the nuanced interplay between molecular structure, binding site adaptability, and cellular determinants of drug response.

### 2.3. Docking Analysis—In Silico Modelling of the Interactions Between the Novel Ferrocene Derivatives and c-Abl Tyrosine Kinase

The ligand efficiencies (LEs), calculated as the ratio of pIC_50_ to the number of heavy atoms in each molecule, are also presented in Table 1. LEs of the studied compounds vary between 0.087 and 0.156, while for imatinib values are in the range of 0.117 and 0.170. As can be seen from Table 1, derivatives **14** and **18** possess higher LEs for cell lines BV-173 and AR-230 (highlighted in bold in Table 1), compared to the control.

Compounds **14** and **18** demonstrated notable activity and higher ligand efficiencies (LEs) against BV-173 and AR-230 cell lines when compared to imatinib. In contrast, compounds **6** and **9** exhibited superior antiproliferative activity against the K-562 cell line. These cell lines are characterized by the expression of the BCR-ABL1 fusion protein, a hallmark of chronic myeloid leukemia (CML) and some cases of acute lymphoblastic leukemia (ALL).

The BCR-ABL1 fusion gene arises from a reciprocal translocation between chromosomes 9 and 22, known as the Philadelphia chromosome (t(9;22)(q34;q11))**.** This translocation results in the fusion of the BCR (breakpoint cluster region) gene on chromosome 22 with the ABL1 (Abelson murine leukemia viral oncogene homolog 1) gene on chromosome 9 [20]. The resultant chimeric protein possesses constitutive tyrosine kinase activity, which disrupts normal cellular signaling and promotes uncontrolled cell proliferation, ultimately contributing to leukemogenesis [21].

**Figure 3:**
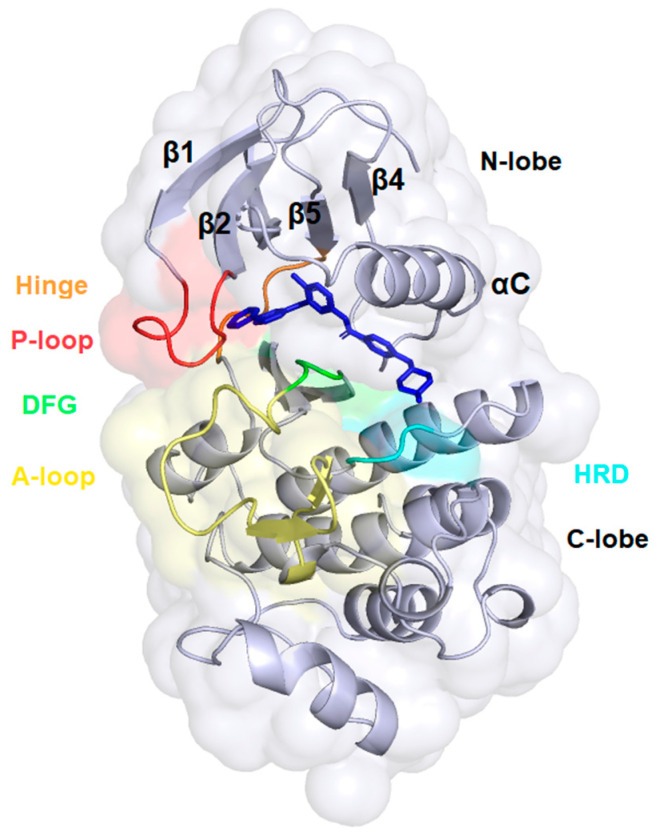
The ATP binding site of cAbl tyrosine kinase in complex with imatinib (PDB ID: 2HYY) [23]. The main segments are depicted as follows: the P-loop in red, the hinge region in orange, the HRD motif in cyan, and the DFG motif in green, which is located at the beginning of the A-loop, represented in yellow. The molecule of imatinib is presented in dark blue.

We conducted molecular docking calculations of the newly synthesized ferrocene derivatives against a potential target we hypothesized—namely, the BCR-ABL1 fusion protein—to investigate their interactions with human cAbl tyrosine kinase (cAbl TK). The cAbl TK consists of two lobes: a smaller N-lobe containing five β-strands (β1–β5) and a single αC-helix (αC), and a larger C-lobe that is primarily α-helical (Figure 3). The ATP-binding site, a deep hydrophobic cavity, is in the cleft between these two lobes. A flexible hinge region connects the lobes and regulates the kinase domain’s opening and closing [20]. Several critical structural elements surround the ATP-binding site, including the P-loop, αC-helix, hinge region, HRD motif, and DFG motif (Figure 3). The P-loop, located between β1 and β2, binds phosphates, while the αC-helix functions as an allosteric element critical for maintaining the kinase’s active conformation and forms part of the regulatory R-spine, a stack of four hydrophobic residues (RS1–RS4), with Met290 serving as RS3. The active state is stabilized by a salt bridge between Glu286 on the αC-helix and Lys271 on β3. The HRD motif (His361–Arg362–Asp363) within the catalytic loop supports the activation loop (A-loop) and contributes ATP binding, while the DFG motif (Asp381–Phe382–Gly383) at the A-loop’s N-terminus helps position the loop and coordinates a magnesium ion necessary for substrate binding and phosphate transfer [20,22]. In the imatinib-bound complex (Figure 3), the DFG motif adopts a flipped DFG-out conformation, while the αC-helix remains in its active (C-in) position, stabilized by Met290 occupying the RS3 pocket and the preserved Glu286–Lys271 salt bridge.

The novel ferrocene derivatives were subject to molecular docking into the ATP binding site of the human cAbl TK domain (PDB 2HYY) [23] utilizing Gnina 1.0 [24]. The predicted affinities and CNN affinities of the ferrocene-containing analogs are presented in Table 2.

**Figure 4:**
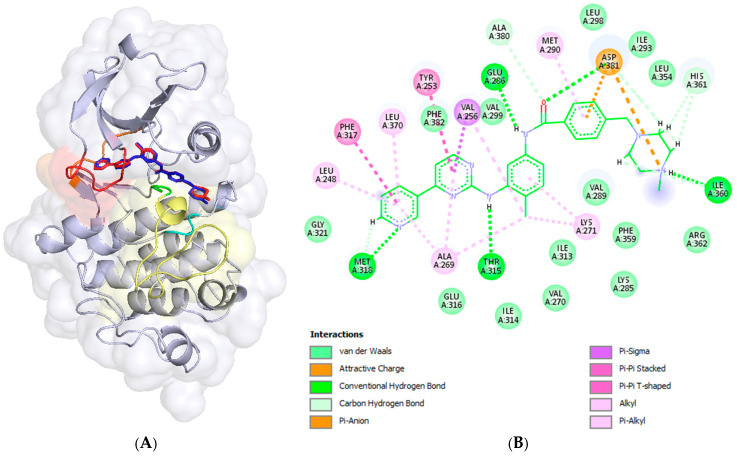

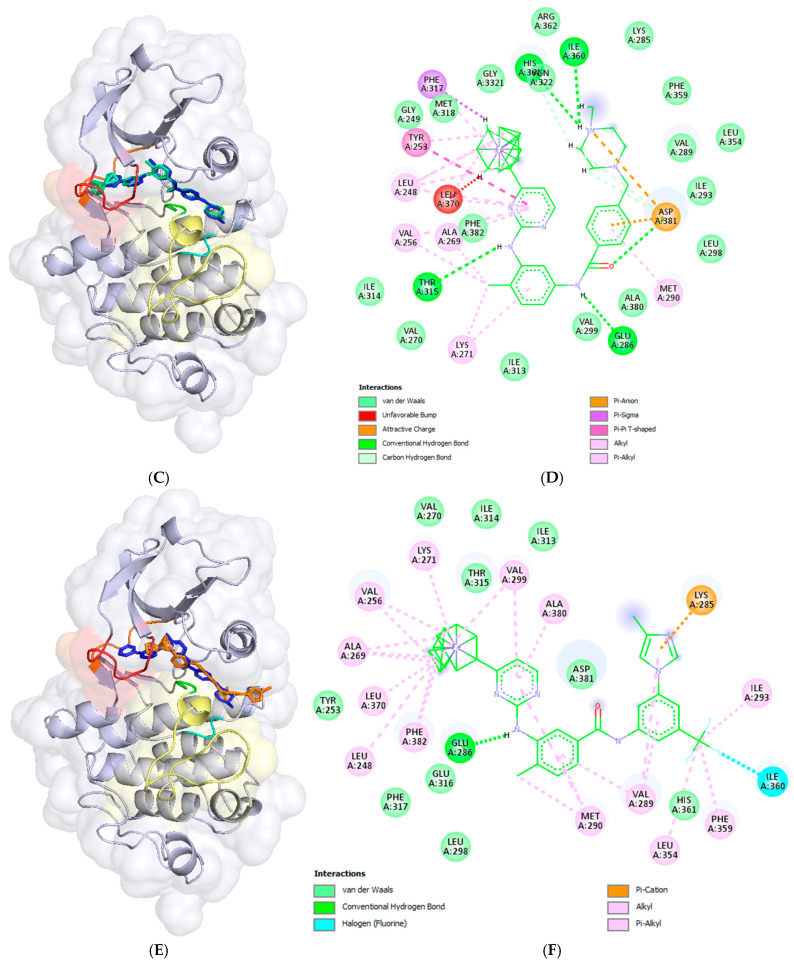

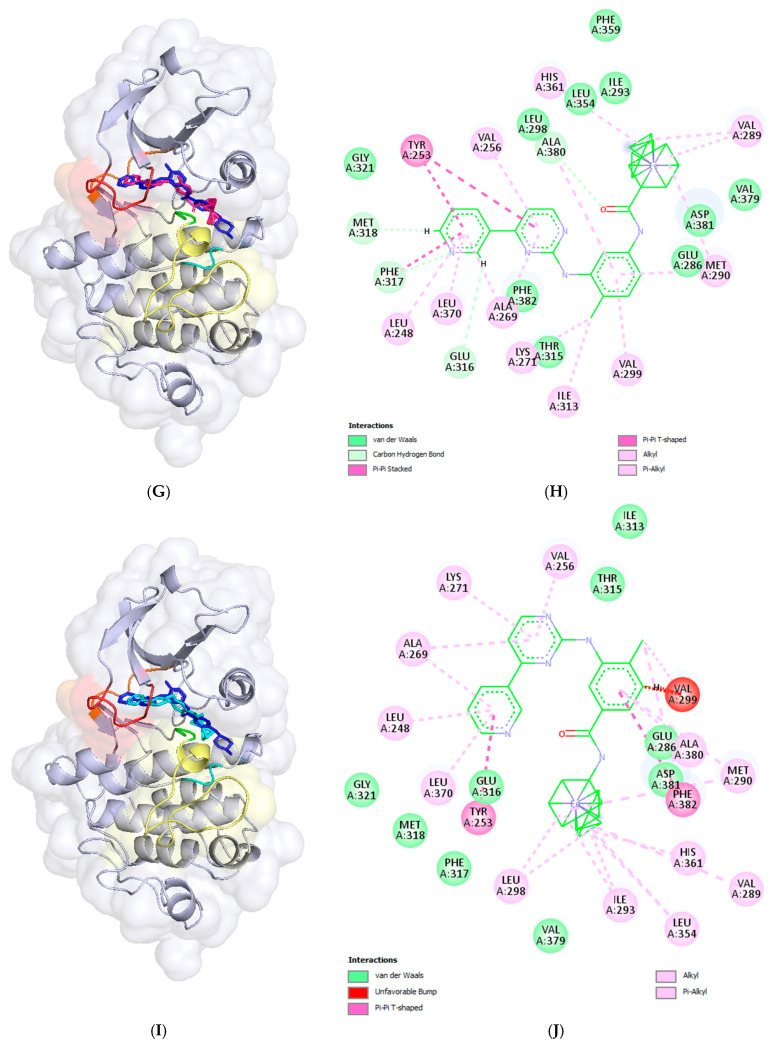
The resulting docking poses (left panels) and intermolecular interactions (right panels) of imatinib (**A**,**B**) and the studied compounds **6** (**C**,**D**), **9** (**E**,**F**), **14** (**G**,**H**), and **18** (**I**,**J**) within the binding site of c-Abl tyrosine kinase are illustrated. The docking complexes in the left panels are superimposed on the crystallographic structure (PDB ID: 2HYY) [23] of the c-Abl TK protein complexed with imatinib (depicted in dark blue). The docked poses of the ligands are color coded as follows: imatinib in red, compound **6** in green, compound **9** in orange, compound **14** in deep pink, and compound **18** in cyan. The binding site segments are colored as in the previous figure: the P-loop in red, the hinge region in orange, the HRD motif in cyan, and the DFG motif in green, located at the beginning of the A-loop, which is represented in yellow. The left panels were prepared using the PyMOL Molecular Graphics System (Version 3.0 Schrödinger, LLC, New York, NY, USA), while the right panels, the 2D diagrams, were generated using BIOVIA, Dassault Systèmes, Discovery Studio Visualizer (v21.1.0.20298, Dassault Systèmes, San Diego, CA, USA, 2021).

The docked compound poses were aligned with the crystallographic structure of imatinib (depicted in dark blue) bound to human c-Abl TK (PDB ID: 2HYY [1]), as shown in the left panels of Figure 4. The corresponding intermolecular interactions within the formed complexes are displayed in the right panels of Figure 4.

The predicted affinity and RMSD value of the re-docked imatinib were 13.14 kcal/mol and 0.206 Å, respectively (Table 2 and Figure 4A). The intermolecular interactions of imatinib within the ATP binding site (BS) of c-Abl TK are well known and studied. It is stabilized within ATP BS through five hydrogen bonds (H-bonds), one ion–ion interaction, and one π-anion contact between the negatively charged carboxyl group of the Asp381 side chain from the DFG motif and the positively charged NMe group of the piperazine ring and the benzamide ring, respectively. Additionally, there are two π-π stackings involving the pyridine and pyrimidine systems with Phe317 in the hinge region and Tyr253 in P-loop, respectively, as well as numerous van der Waals interactions (Figure 4B). The hydrogen bonds were formed between the pyridine nitrogen atom and the backbone amino hydrogen atom of Met318 in the hinge region; the hydrogen atom of the linker amino group and the oxygen atom of the side chain hydroxyl group of the gatekeeper residue Thr315 from the hinge region; the hydrogen and oxygen atoms of the linker amide group and the side chain carboxy group of Glu286 from the αC-helix in the N-lobe; the backbone NH group of Asp381 from the DFG motif; and the hydrogen atom at the quaternary NMe group of piperazine and the backbone carbonyl oxygen atom of Ile360 adjacent to the HRD motif.

The substitution of the pyridine group with a ferrocene moiety in compound **6** resulted in the loss of one hydrogen bond present in imatinib, specifically the bond between the pyridine nitrogen atom and Met318. However, an additional hydrogen bond was gained between the hydrogen atom of the quaternary NMe group on the piperazine ring and the backbone carbonyl oxygen atom of His360 from the HRD motif (Figure 4C,D). As shown in Figure 4C, although the predicted pose of compound **6** differs slightly from the crystallographic pose of imatinib, the retained imatinib portion of the molecule engages in the same interactions as previously described for imatinib itself. The ferrocene moiety in compound **6** participates in numerous van der Waals interactions; however, an unfavorable steric clash occurs with Leu370 from the P-loop. This could be attributed to the rigidity of the binding site during molecular docking calculations.

The docked pose of compound **14**, in which the piperazine and benzamidine parts of imatinib were replaced with a ferrocene moiety, is shown in Figure 4G. It can be observed that the ferrocene (Fc) moiety is well inserted into the hydrophobic pocket, accompanied by a significant shift in the phenyl ring. This results in the loss of all hydrogen bonds present in the imatinib complex, as well as the ion–ion and π-anion interactions observed at the substituted structural segments of imatinib (Figure 4G,H). However, the formed complex is stabilized by an additional π-π stacking interaction between the pyridine ring and Tyr253, along with numerous van der Waals interactions.

The docking pose of compound **9**, a structural analogue of nilotinib with a pyridine ring replaced by a ferrocenyl moiety (Figure 2), indicates that the compound is incompletely inserted within the deep binding pocket of c-Abl TK. This incomplete insertion prevents the compound from reaching Met318 in the hinge region, resulting in significant solvent exposure of the entire methyl group at the imidazole ring, as well as half of the imidazole ring (Figure 4C,D). The ferrocene derivative forms one hydrogen bond between the hydrogen atom of the amino group in the linker and the side chain carboxyl group of Glu286 from the αC-helix in the N-lobe (Figure 4D). The imidazole π-system participates in a π-cation interaction with the protonated side chain amino group of Lys285 from the αC-helix. Additionally, numerous van der Waals interactions help stabilize the formed complex (Figure 4D).

In the case of the resulting docking pose of compound **18**, an Fc-analogue of nilotinib in which the trifluoromethylphenyl and imidazole systems were replaced with a ferrocene moiety (Figure 2), the pyridine ring inserts deeply into the binding site but does not make contact with Met318 in the hinge region (Figure 4I,J). As a result, the rest of the molecule is oriented and shifted differently, causing the loss of the hydrogen bond with Thr315. Two π-π stacking interactions are formed: one between the pyridine system and Tyr253, and another between the phenyl ring and Phe382 from the DFG motif in the A-loop. An unfavorable clash occurs between the phenyl ring and Val299, likely due to the rigid binding site during the docking calculations. Numerous van der Waals interactions help stabilize the formed complex.

### 2.4. Comparative Analysis Between Experimental and Predicted Ligand Efficiencies

LE_AFF_ and LE_CNN_ were calculated similarly to LEs (Table 2). For affinity, the absolute values were considered as a more negative affinity value indicates stronger binding, greater inhibition, and consequently, enhanced anticancer activity.

**Figure 5:**
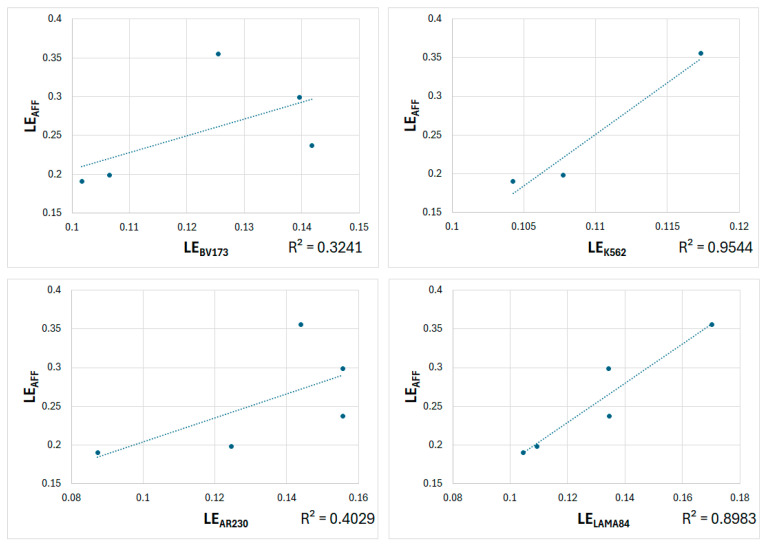
Plots of predicted LE_AFF_ vs experimental LEs (LE_BV173_, LE_K562_, LE_AR230_, and LE_LAMA84_).

**Figure 6:**
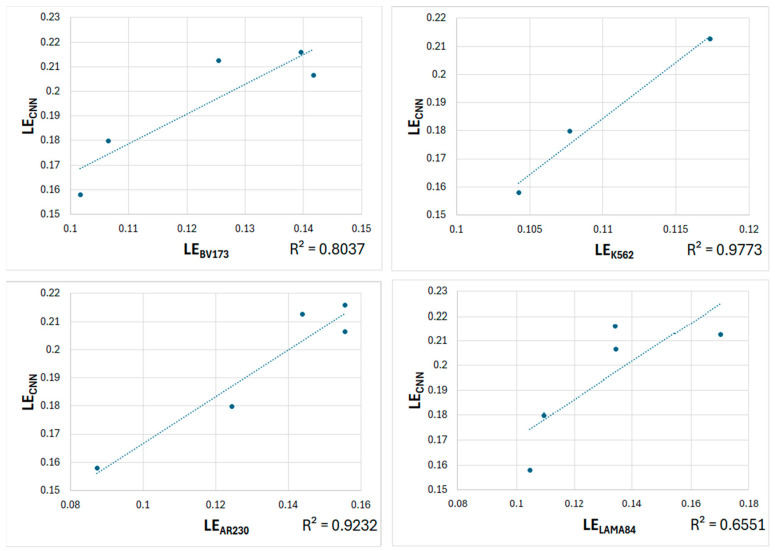
Plots of predicted LE_CNN_ vs. experimental LEs (LE_BV173_, LE_K562_, LE_AR230_, and LE_LAMA84_).

Since LE values reflect affinities per atom and are considered more useful for ligand optimization than directly comparing experimental biological activities between compounds [25], we decided to correlate the LE values derived from experimental data (Table 1) with those obtained from docking predictions (Table 2). The corrections are provided in Table 3 and Figure 5 and Figure 6.

Table 3 shows that the correlations between the predicted LE_AFF_ and two experimental values, LE_BV173_ and LE_AR230_, are moderate (0.569 and 0.635, respectively). However, the correlations with the other two experimental values, LE_K562_ and LE_LAMA84_, are excellent (0.977 and 0.948, respectively). For LE_CNN_, the correlations with all experimental values are excellent, ranging from 0.896 to 0.989, although the correlation for LE_LAMA84_ is considered good (0.809). These trends are clearly shown in the corresponding graphs in Figure 6.

The relationships between the predicted and experimental LEs suggest that c-Abl tyrosine kinase may be a potential target for the anticancer activity of the newly synthesized Fc-analogs of imatinib and nilotinib.

## 3. Materials and Methods

### 3.1. Synthesis

#### 3.1.1. General

Reagents were commercial grade and used without further purification. Thin layer chromatography (TLC) was performed using aluminum sheets pre-coated with silica gel 60 F254 (Merck, Darmstadt, Germany). Flash column chromatography was carried out using Silica Gel 60 230–400 mesh (Acros Organics, Beijing, China). Commercially available solvents were used for reactions, TLC, and column chromatography. Melting points were determined in a capillary tube on BUCHI Melting Point B-535 Apparatus 220v (BÜCHI Labortechnik AG, Flawil, Switzerland, uncorrected). The NMR spectra were recorded on a Bruker Avance NEO 400 MHz (400.13 for ^1^H NMR and 100.6 MHz for ^13^C NMR) spectrometer (Bruker, Berlin, Germany) with TMS as an internal standard for chemical shifts. ^1^H and ^13^C NMR data are reported as follows: chemical shift, multiplicity (s = singlet, d = doublet, t = triplet, q = quartet, br = broad, and m = multiplet), coupling constants (Hz), integration. The high resolution mass spectra (HRMS) of the compounds were recorded on a Thermo Scientific Q Exactive Plus Hybrid Quadrupole-Orbitrap Mass Spectrometer (Thermo Fisher Scientific, Bremen, Germany). MS acquisition was carried out with a heated electrospray ionization (HESI) in positive mode.

#### 3.1.2. Benzyl (4-Methyl-3-nitrophenyl)carbamate (**1a**)

To a stirred solution of 4-methyl-3-nitroaniline (0.609 g, 4.0 mmol) in THF (16 mL) were added at 0 °C sat. aq. NaHCO_3_ (5 mL) and benzyl chloroformate (0.72 mL, 8.0 mmol). The mixture was stirred overnight at r.t., diluted with water (15 mL) and extracted with ethyl acetate. The combined organic layers were washed with brine, dried over anhydrous MgSO_4_, filtered, and concentrated under reduced pressure. The crude product was purified by flash column chromatography (silica gel, petroleum ether/EtOAc = 4:1). Yield 99%. ^1^H NMR (CDCl_3_, 400 MHz): δ 8.07 (d, *J* = 2.3 Hz, 1H), 7.57 (d, *J* = 7.7 Hz, 1H), 7.43–7.36 (m, 5H), 7.2 (d, *J* = 8.3 Hz, 1H), 6.84 (brs, 1H, NH), 5.24 (s, 2H), 2.56 (s, 3H) ppm. ^13^C NMR (CDCl_3_, 100.6 MHz): δ 153.07 (C), 149.25 (C), 136.74 (CH), 135.61 (CH), 134.8 (C), 133.26 (CH), 128.71 (2 CH), 128.58 (CH), 128.42 (2 CH), 128.21 (C), 122.94 (C), 67.49 (CH_2_), 19.84 (CH_3_) ppm.

#### 3.1.3. Benzyl (3-Amino-4-methylphenyl)carbamate (**2a**)

To a solution of nitro compound **1a** (1.135 g, 4 mmol) in ethanol (60 mL) was added Fe dust (0.890 g, 16.0 mmol). The suspension was heated to reflux and aq. NH_4_Cl (2.140 g in 12 mL, 40.00 mmol) was added dropwise at this temperature. After complete addition, the mixture was refluxed for an additional 1.5 h, then cooled to r.t., filtered through folded filter paper, and washed with ethanol. The filtrate was concentrated under reduced pressure. The residue was dissolved in CH_2_Cl_2_ and washed with water. The organic layer was dried over MgSO_4_, filtered, and concentrated under reduced pressure. The residue was washed with petroleum ether/Et_2_O = 1:1. Yield 82%. ^1^H NMR (CDCl_3_, 400 MHz): δ 7.41–7.33 (m, 5H), 6.95 (s, 1H), 6.93 (s, 1H, NH), 6.54 (dd *J* = 8.0; 2.2 Hz, 2H), 5.18 (s, 2H), 3.62 (s, 2H, NH_2_), 2.11 (s, 3H) ppm. ^13^C NMR (CDCl_3_, 100.6 MHz): δ 153.36 (C), 145.15 (CH), 136.68 (C), 136.21 (CH), 130.69 (CH), 128.62 (2 CH), 128.12 (CH), 128.29 (2CH), 117.60 (C), 108.86 (C), 105.32 (C), 66.89 (CH_2_), 16.75 (CH_3_) ppm.

#### 3.1.4. Benzyl (3-Guanidino-4-methylphenyl)carbamate Hydrochloride (**3a**)

To an ice-cold solution of **2a** (0.845 g, 3.300 mmol) in Et_2_O was added dropwise HCl (2N solution in Et_2_O, 1.73 mL, 3.465 mmol). The mixture was stirred for 0.5 h at r.t. and then concentrated under reduced pressure. The obtained hydrochloride was slowly added under stirring to molten cyanamide (0.555 g, 13.200 mmol) at 50 °C. The temperature was then raised to 65–70 °C and the molt was gently stirred for 1.5 h. The reaction was then cooled to r.t., Et_2_O was added to the oily residue, and the mixture was vigorously stirred for 30 min. The crude precipitate was filtered off, washed with Et_2_O, and dried under vacuum. Yield 99%. ^1^H NMR (DMSO-*d*_6_, 400 MHz): δ 9.89 (s, 1H, NH), 9.72 (s, 1H, NH), 7.42–7.37 (m, 10H), 7.25–7.23 (m, 1H), 5.16 (s, 2H), 2.15 (s, 3H) ppm. ^13^C NMR (DMSO-*d*_6_, 100.6 MHz): δ = 156.87 (C), 153.76 (C), 138.51 (C), 137.02 (CH), 133.78 (CH), 131.72 (CH), 128.93 (2 CH), 128.58 (2 CH), 128.55 (CH), 117.97 (C), 117.14 (C), 66.25 (CH_2_), 17.18 (CH_3_) ppm.

#### 3.1.5. Benzyl (4-Methyl-3-((4-ferrocenylpyrimidin-2-yl)amino)phenyl)carbamate (**4a**)

To a solution of **3a** (0.408 g, 1.270 mmol) and K_2_CO_3_ (0.440 g, 3.180 mmol) in *n*-butanol (15 mL) was added *(E)-3-(Dimethylamino)-1-ferrocenyl-2-propen-1-one* (0.300 g, 1.060 mmol). The mixture was refluxed for 48 h, cooled to r.t., and filtered through a pad of silica gel (EtOAc). The filtrate was concentrated under reduced pressure and subjected to purification by flash column chromatography (silica gel, EtOAc/CH_3_OH = 100:1). Yield 55%. ^1^H NMR (CDCl_3_, 400 MHz): δ 8.27 (brs, 1H), 8.24 (d, *J* = 5.3 Hz, 1H, CH), 7.37–7.36 (m, 5H), 7.31–7.28 (m, 1H, CH), 7.14–7.11 (m, 1H, CH), 6.96 (brs, 1H, HNCO), 6.78 (d, *J* = 5.3 Hz, 1H, CH), 6.65 (brs, 1H, NH), 4.97 (t, *J* = 1.9 Hz, 2H, Cp-H), 4.70 (s, 2H, CH_2_), 4.47 (t, *J* = 1.9 Hz, 2H, Cp-H), 4.10 (s, 5H, Cp5), 2.31 (s, 3H, CH_3_) ppm. ^13^C NMR (CDCl_3_, 100.6 MHz): δ 168.94 (C), 160.04 (C), 156.78 (CH), 140.90 (C), 138.29 (C), 136.40 (C), 130.64 (CH), 128.57 (2 CH), 127.65 (3 CH), 123.69 (C), 113.52 (CH), 111.59 (CH), 108.59 (CH), 80.59 (C), 70.98 (2 CH), 70.03 (5 CH), 68.17 (2 CH), 65.36 (CH_2_), 17.57 (CH_3_) ppm.

#### 3.1.6. *N*-(4-Methyl-3-nitrophenyl)acetamide (**1b**)

To a solution of 4-methyl-3-nitroaniline (1.000 g, 6.57 mmol) and NEt_3_ (1.83 mL, 13.14 mmol) in CH_2_Cl_2_ (30 mL) was added dropwise acetic anhydride (0.93 mL, 9.86 mmol) at 0 °C. The reaction was stirred at r.t. for 3.5 h and quenched with sat. aq. NaHCO_3_. The mixture was extracted with CH_2_Cl_2_. The combined organic layers were washed with 2N HCl followed by water, dried over MgSO_4_, filtered, and concentrated under reduced pressure. The residue was washed with petroleum ether/EtOAc = 10:1. Yield 91%. ^1^H NMR (CDCl_3_, 400 MHz): δ 8.13 (d, *J* = 1.9 Hz, 1H), 7.77 (dd, *J* = 8.4; 1.9 Hz, 1H), 7.63 (brs, 1H, NH), 7.30 (d, *J* = 8.4 Hz, 1H), 2.57 (s, 3H, CH_3_), 2.23 (s, 3H, CH_3_) ppm. ^13^C NMR (CDCl_3_, 100.6 MHz): δ 168.68 (C), 149.0 (C), 136.74 (C), 133.22 (CH), 129.07 (C), 124.32 (CH), 115.66 (CH), 24.51 (CH_3_), 19.95 (CH_3_) ppm.

#### 3.1.7. *N*-(3-Amino-4-methylphenyl)acetamide (**2b**)

To a solution of nitro compound **1b** (0.585 g, 3.0 mmol) in ethanol (50 mL) was added Fe dust (0.670 g, 12.0 mmol). The suspension was heated to reflux and aq. NH_4_Cl (1.605 g in 9 mL, 30.00 mmol) was added dropwise at this temperature. After complete addition, the mixture was refluxed for 1.5 h, then cooled to r.t., filtered through folded filter paper, and washed with ethanol. The filtrate was concentrated under reduced pressure, dissolved in CH_2_Cl_2_, dried over MgSO_4_, filtered, and concentrated under reduced pressure. Yield 93%. ^1^H NMR ((DMSO-*d*_6_, 400 MHz): δ 9.54 (s, 1H, NH), 6.93 (d, *J* = 1.9 Hz, 1H), 6.79 (d, *J* = 8.0 Hz, 1H), 6.62 (dd, *J* = 8.0; 1.9 Hz, 1H), 4.78 (brs, 1H, NH_2_), 1.98 (s, 6H, 2 CH_3_) ppm. ^13^C NMR ((DMSO-*d*_6_, 100.6 MHz): δ 168.18 (C), 146.96 (C), 138.28 (C), 130.16 (CH), 116.50 (C), 107.84 (CH), 105.46 (CH), 24.43 (CH_3_), 17.37 (CH_3_) ppm.

#### 3.1.8. *N*-(3-Guanidino-4-methylphenyl)acetamide Hydrochloride (**3b**)

To an ice-cold solution of **2b** (0.456 g, 2.77 mmol) in CH_2_Cl_2_ was added dropwise HCl (2N solution in Et_2_O, 1.53 mL, 3.05 mmol). The mixture was stirred for 0.5 h at r.t. and then concentrated under reduced pressure. The obtained hydrochloride was slowly added under stirring to molten cyanamide (0.466 g, 11.08 mmol) at 50 °C. The temperature was then raised to 65–70 °C and the melt was gently stirred for an additional 2 h. The reaction mixture was then cooled to r.t., Et_2_O was added to the oily residue, and the mixture was vigorously stirred for 30 min. The crude precipitate was filtered off, washed with Et_2_O, and dried under vacuum. Yield 97%. ^1^H NMR (DMSO-*d*_6_, 400 MHz): δ 10.17 (s, 1H, NH), 9.68 (s, 1H, NH), 7.55 (d, *J* = 1.5 Hz, 1H), 7.47 (dd, *J* = 8.3; 1.8 Hz, 1H), 7.37 (s, 3H), 7.24 (d, *J* = 8.3 Hz, 1H), 2.15 (s, 3H, CH_3_), 2.05(s, 3H, CH_3_) ppm. ^13^C NMR (DMSO-*d*_6_, 100.6 MHz): δ 168.84 (C), 156.85 (C), 138.80 (C), 133.56 (C), 131.58 (CH), 129.54 (C), 118.78 (CH), 117.97 (CH), 24.44 (CH_3_), 17.26 (CH_3_) ppm.

#### 3.1.9. *N*-(4-Methyl-3-((4-ferrocenylpyrimidin-2-yl)amino)phenyl)acetamide (**4b**)

To a solution of **3b** (0.309 g, 1.270 mmol) and K_2_CO_3_ (0.440 g, 3.180 mmol) in *n*-butanol (15 mL) was added *(E)-3-(Dimethylamino)-1-ferrocenyl-2-propen-1-one* (0.300 g, 1.060 mmol). The mixture was refluxed for 48 h, cooled to r.t., and filtered through a pad of silica gel (EtOAc). The filtrate was concentrated under reduced pressure and subjected to purification by flash column chromatography (silica gel, CH_2_Cl_2_/CH_3_OH = 50:1). Yield 41%. ^1^H NMR (CDCl_3_, 400 MHz): δ 8.27–8.25 (m, 2H), 7.46 (brs, 1H, NH), 7.31 (dd, *J* = 8.2; 1.7 Hz, 1H), 7.16 (d, *J* = 8.2 Hz, 1H), 6.91 (s, 1H, NH), 6.81 (d, *J* = 5.2 Hz, 1H), 4.98 (t, *J* = 1.8 Hz, 2H, Cp-H), 4.50 (t, *J* = 1.8 Hz, 2H, Cp-H), 4.12 (s, 5H, Cp5), 2.34 (s, 3H, CH_3_), 2.15 (s, 3H, CH_3_) ppm. ^13^C NMR (CDCl_3_, 100.6 MHz): δ 168.99 (C), 168.18 (C), 160.14 (C), 156.64 (CH), 138.15 (C), 136.44 (C), 130.67 (CH), 123.82 (C), 114.95 (CH), 112.74 (CH), 108.71 (CH), 80.59 (C), 70.99 (2 CH), 70.03 (5 CH), 68.12 (2 CH), 24. 55 (CH_3_), 17.68 (CH_3_) ppm.

#### 3.1.10. 6-Methyl-N^1^-(4-ferrocenylpyrimidin-2-yl)benzene-1,3-diamine (**5**)

To a stirred solution of **4b** (0.138 g, 0.324 mmol) in EtOH (5 mL) was added hydrazine hydrate (2 mL). The solution was heated at 75 °C for 72 h. The reaction mixture was cooled to r.t. dissolved in EtOAc and washed with water. The organic layer was dried over MgSO_4_, filtered, and concentrated under reduced pressure. The crude product was purified by flash column chromatography (silica gel, CH_2_Cl_2_/CH_3_OH = 50:1). Yield 36%. ^1^H NMR (CDCl_3_, 400 MHz): δ 8.25 (d, *J* = 5.2 Hz, 1H), 7.79 (d, *J* = 2.3 Hz, 1H), 6.99 (d, *J* = 8.0 Hz, 1H), 6.87 (brs, 1H, NH), 6.77 (d, *J* = 5.2 Hz, 1H), 6.38 (dd, *J* = 8.0; 2.3 Hz, 1H), 4.95 (t, *J* = 1.9 Hz, 2H, Cp-H), 4.47 (t, *J* = 1.9 Hz, 2H, Cp-H), 4.10 (s, 5H, Cp5), 3.68 (brs, 1H, NH_2_), 2.26 (s, 3H, CH_3_) ppm. ^13^C NMR (CDCl_3_, 100.6 MHz): δ 168.60 (C), 160.14 (C), 156.86 (CH), 145.01 (C), 138.60 (C), 130.84 (CH), 117.34 (C), 109.76 (CH), 107.75 (CH), 80.77 (C), 70.88 (2 CH), 69.87 (5 CH), 68.04 (2 CH), 17.26 (CH_3_) ppm.

#### 3.1.11. *N*-(4-Methyl-3-((4-ferrocenylpyrimidin-2-yl)amino)phenyl)-4-((4-methylpiperazin-1-yl)methyl)benzamide (**6**)

4-[(4-methylpiperazin-1-yl)methyl]benzoic acid dihydrochloride (0.58 g, 0.187 mmol) was added at r.t. to a solution of CDI (0.029 g, 0.179 mmol) in dry DMF (2 mL) under an argon atmosphere. The mixture was stirred for 2 h at 50 °C, and a solution of **5** (0.060 g, 0.156 mmol) in dry DMF (1 mL) was added. The reaction mixture was stirred at 70 °C for 20 h, cooled to r.t., diluted with H_2_O and extracted with CH_2_Cl_2_. The combined organic layers were washed with brine, dried over MgSO_4_, filtered, and concentrated under reduced pressure. The crude product was purified by flash column chromatography (silica gel, CH_2_Cl_2_/CH_3_OH/NH_4_OH = 10:1:0.1). Yield 59%. Mp 154–156 °C. ^1^H NMR (CDCl_3_, 400 MHz): δ 8.53 (d, *J* = 2.1 Hz, 1H), 8.26 (d, *J* = 5.2 Hz, 1H), 7.91 (brs, 1H, HNCO), 7.84 (d, *J* = 8.0 Hz, 2H), 7.45–7.41 (m, 3H), 7.19 (d, *J* = 8.2 Hz, 1H), 6.89 (s, 1H, NH), 6.79 (d, *J* = 5.2 Hz, 1H), 4.95 (t, *J* = 1.9 Hz, 2H, Cp-H), 4.46 (t, *J* = 1.9 Hz, 2H, Cp-H), 4.09 (s, 5H, Cp5), 3.58 (s, 2H, NCH_2_), 2.55 (brs, 8H, CH_2_), 2.36 (s, 3H, NCH_3_), 2.35 (s, 3H, CH_3_) ppm. ^13^C NMR (CDCl_3_, 100.6 MHz): δ 168.84 (C), 167.50 (CO), 160.04 (C), 156.80 (CH), 142.17 (C), 138.35 (C), 136.41 (C),134.01 (C), 130.69 (CH), 129.29 (2 CH), 127.04 (2 CH), 123.58 (C), 114.83 (CH), 112.56 (CH), 108.66 (CH), 80.60 (C), 70.93 (2 CH, Cp), 69.99 (5 CH, Cp5), 68.10 (2 CH, Cp), 54.88 (4 CH_2_), 52.55 (4 CH_2_), 45.62 (NCH_3_), 17.68 (CH_3_) ppm. HRMS (HESI): found for C_34_H_36_FeN_6_O [M + H]^+^ *m*/*z* 601.2373, calcd. *m*/*z* 601.2300.

#### 3.1.12. 4-Methyl-*N*-(3-(4-methyl-1H-imidazol-1-yl)-5-(trifluoromethyl)phenyl)-3-((4-ferrocenylpyrimidin-2-yl)amino)benzamide (**9**)

A solution of 4-methyl-3-((4-ferrocenylpyrimidin-2-yl)amino)benzoic acid **7** (0.120 g, 0.290 mmol), DIPEA (0.1 mL, 0.580 mmol), and HATU (0.110 g, 0.290 mmol) in dry DMF (3 mL) was stirred at r.t. for 15 min under an argon atmosphere. 3-(4-Methyl-1H-imidazol-1-yl)-5-(trifluoromethyl)aniline **8** (0.077 g, 0.319 mmol) was added and the reaction was stirred at 50 °C for 5 days. After cooling to r.t., the mixture was diluted with H_2_O and extracted with CH_2_Cl_2_. The combined organic layers were washed with brine, dried over MgSO_4_, and concentrated. The crude product was purified by flash column chromatography (silica gel, CH_2_Cl_2_/CH_3_OH/NH_4_OH = 30:1:0.1). Yield 39%. Mp 111–113 °C. ^1^H NMR (CDCl_3_, 400 MHz): δ 9.02 (s, 1H, NH), 8.87 (d, *J* = 1.1 Hz, 1H), 8.19 (d, *J* = 5.3 Hz, 1H), 7.78 (brs, 1H), 7.73 (d, *J* = 1.0 Hz, 1H), 7.48 (dd, *J* = 7.8; 1.8 Hz, 1H), 7.25–7.23 (m, 2H), 6.99 (s, 1H), 6.93 (s, 1H, NH), 6.76 (d, *J* = 5.3 Hz, 1H), 4.89 (t, *J* = 1.9 Hz, 2H, Cp-H), 4.38 (t, *J* = 1.9 Hz, 2H, Cp-H), 4.01 (s, 5H, Cp5), 2.34 (s, 3H, CH_3_), 2.20 (d, *J* = 0.7 Hz, 3H, CH_3_) ppm. ^13^C NMR (CDCl_3_, 100.6 MHz): δ 169.19 (C), 169.56 (C), 159.66 (C), 155.78 (CH), 140.88 (C), 140.02 (C), 138.36 (C), 138.28 (C), 134.51 (CH), 132.57 (C), 131.20 (C), 130.87 (CH), 121.58 (CH), 118.31 (CH), 115.13 (CH), 114.75 (CH), 114.38 (CH), 112.40 (CH), 109.08 (CH), 80.26 (C), 71.25 (2 CH, Cp), 70.08 (5 CH, Cp5), 67.98 (2 CH, Cp), 18.21 (CH_3_), 13.57 (CH_3_) ppm. ^19^F NMR (CDCl_3_,): δ −62.79. HRMS (HESI): found for C_33_H_27_F_3_FeN_6_O [M + H]^+^ *m*/*z* 637.1620, calcd. *m*/*z* 637.1548.

#### 3.1.13. 1-(2-Methyl-5-nitrophenyl)guanidine Hydrochloride (**11**)

To an ice-cold solution of 2-methyl-5-nitroaniline (2.00 g, 13.14 mmol) in Et_2_O (35 mL) was added dropwise HCl (2N solution in Et_2_O, 7.00 mL, 14.00 mmol). The mixture was stirred for 0.5 h at r.t. and then concentrated under reduced pressure. The obtained hydrochloride was slowly added under stirring to molten cyanamide (1.64 g, 39.00 mmol) at 50 °C. The temperature was then raised to 65–70 °C and the melt was gently stirred for an additional 2 h. The reaction was then cooled to r.t., Et_2_O was added to the oily residue, and the mixture was vigorously stirred for 30 min. The crude precipitate was filtered off, washed with Et_2_O and dried under vacuum. Yield 94%. ^1^H NMR (DMSO-*d*_6_, 400 MHz): δ 8.21 (d, *J* = 2.4 Hz, 1H), 8.19 (d, *J* = 2.4 Hz, 1H), 8.16 (d, *J* = 2.3 Hz, 2H), 7.63 (d, *J* = 8.5 Hz, 2H), 2.43(s, 3H, CH_3_) ppm. ^13^C NMR (DMSO-*d*_6_, 100.6 MHz): δ 158.35 (C), 148.44 (C), 145.36 (C), 135.34 (C), 133.54 (CH), 124.41 (CH), 124.20 (CH), 17.97 (CH_3_) ppm.

#### 3.1.14. N-(2-Methyl-5-nitrophenyl)-4-(pyridin-3-yl)pyrimidin-2-amine (**12**)

To a solution of **11** (2.07 g, 9.00 mmol) in *n*-butanol (10 mL) was added *(E)-3-(dimethylamino)-1-(pyridin-3-yl)prop-2-en-1-one* **10** (1.22 g, 6.94 mmol) and NaOH (0.390 g, 9.72 mmol), and the suspension was refluxed for 16 h. After cooling to r.t., a precipitate was formed. It was filtered off, suspended in H_2_O and vigorously stirred for 1 h. The product was collected on a Schott filter, washed with H_2_O and CH_3_OH and dried. Yield 70%. ^1^H NMR (DMSO-*d*_6_, 400 MHz): δ 9.32 (d, *J* = 1.1 Hz, 1H), 9.25 (s, 1H, NH), 8.80 (s, 1H), 8.71 (d, *J* = 3.8 Hz, 1H), 8.63 (d, *J* = 5.1 Hz, 1H), 8.48 (d, *J* = 7.9 Hz, 1H), 7.89 (dd, *J* = 8.3; 1.9 Hz, 1H), 7.58 (d, *J* = 5.1 Hz, 1H), 7.55 (dd, *J* = 7.8; 4.9 Hz, 1H), 7.51 (d, *J* = 8.3 Hz, 1H), 2.43 (s, 3H, CH_3_) ppm. ^13^C NMR (DMSO-*d*_6_, 100.6 MHz): δ 162.07 (C), 160.77 (C), 160.18 (CH), 152.12 (CH), 148.65 (CH), 146.28 (C), 139.36 (C), 139.21 (C), 134.79 (CH), 132.35 (C), 131.72 (CH), 124.37 CH), 118.42 (CH), 117.95 (CH), 109.37 (CH), 18.87 (CH_3_) ppm.

#### 3.1.15. 6-Methyl-N^1^-(4-(pyridin-3-yl)pyrimidin-2-yl)benzene-1,3-diamine (**13**)

To a solution of nitro compound **12** (0.100 g, 0.33 mmol) in ethanol (5 mL) was added Fe dust (0.074 g, 1.32 mmol). The suspension was heated to reflux and aq. NH_4_Cl (0.177 g, 3.3 mmol in 1 mL H_2_O) was added dropwise at this temperature. After complete addition, the mixture was refluxed for 2 h, then cooled to r.t., filtered through folded filter paper, and washed with ethanol. The filtrate was concentrated under reduced pressure, dissolved in CH_2_Cl_2_, and washed with water. The organic layer was dried over MgSO_4_, filtered, and concentrated under reduced pressure. The product was purified by filtration through a pad of silica gel (CH_2_Cl_2_/CH_3_OH = 20:1). Yield 99%. ^1^H NMR (CDCl_3_, 400 MHz): δ 9.27 (s, 1H, NH), 8.72 (d, *J* = 3.6 Hz, 1H), 8.50 (d, *J* = 4.7 Hz, 1H), 8.35 (d, *J* = 7.6 Hz, 1H), 7.43 (dd, *J* = 6.8; 4.7 Hz, 1H), 7.26 (s, 1H), 7.15 (d, *J* = 4.9 Hz, 1H), 7.01–6.99 (m, 2H), 6.42 (d, *J* = 7.1 Hz, 1H), 3.49 (s, 2H, NH_2_), 2.26 (s, 3H) ppm. ^13^C NMR (CDCl_3_, 100.6 MHz): δ 162.53 (C), 160.66 (C), 158.99 (CH), 151.41 (CH), 148.53 (CH), 145.08 (C), 137.88 (C), 134.45 (CH), 132.73 (C), 131.03 (CH), 123.61 (CH), 118.11 (C), 110.61 (CH), 108.37 (CH), 108.04 (CH), 17.22 (CH_3_) ppm.

#### 3.1.16. *N*-(4-Methyl-3-((4-(pyridin-3-yl)pyrimidin-2-yl)amino)phenyl)ferrocenecarboxamide (**14**)

A solution of ferrocenecarboxylic acid (0.092 g, 0.400 mmol), DIPEA (0.18 mL, 1.080 mmol), and HATU (0.152 g, 0.400 mmol) in dry DMF (3 mL) was stirred at r.t. for 15 min under an argon atmosphere. Amine **13** (0.100 g, 0.360 mmol) was added and the reaction was stirred at r.t. for 24 h. The mixture was diluted with H_2_O, and the formed precipitate was filtered off and washed with water. It was dissolved in CH_2_Cl_2_/CH_3_OH = 20:1, dried over MgSO_4_ and concentrated under reduced pressure. The product was purified by flash column chromatography (silica gel, CH_2_Cl_2_/CH_3_OH = 20:1). Yield 81%. Mp 238–240 °C. ^1^H NMR (DMSO-*d*_6_, 400 MHz): δ 9.39 (s, 1H, NH), 9.28 (d, *J* = 1.5 Hz, 1H), 8.97 (s, 1H), 8.69 (d, *J* = 5.1 Hz, 1H), 8.52 (d, *J* = 5.1 Hz, 1H), 8.48 (dt, *J* = 8.0; 1.7 Hz, 1H), 8.01 (d, *J* = 1.6 Hz, 1H), 7.52 (dd, *J* = 8.0; 4.8 Hz, 1H), 7.45–7.43 (m, 2H), 7.19 (d, *J* = 8.3 Hz, 1H), 5.01 (t, *J* = 1.8 Hz, 2H, Cp-H), 4.37 (t, *J* = 1.8 Hz, 2H, Cp-H), 4.21 (s, 5H, Cp5), 2.21 (s, 3H, CH_3_) ppm. ^13^C NMR (DMSO-*d*_6_, 100.6 MHz): δ 168.39 (C), 162.06 (C), 161.70 (C), 159.96 (CH), 151.85 (CH), 148.66 (CH), 138.22 (C), 137.73 (C), 134.85 (CH), 132.71 (C), 130.43 (CH), 127.66 (C), 124.26 (CH), 117.75 (CH), 117.25 (CH), 107.95 (CH), 77.05 (C), 70.87 (2 CH, Cp), 69.89 (5 CH, Cp5), 60.05 (2 CH, Cp), 18.09 (CH_3_) ppm. HRMS (HESI): found for C_27_H_23_FeN_5_O [M + H]^+^ *m*/*z* 490.1319, calcd. *m*/*z* 490.1252.

#### 3.1.17. 4-Methyl-3-((4-(pyridin-3-yl)pyrimidin-2-yl)amino)benzoic Acid (**17**)

To a solution of 1-(5-(methoxycarbonyl)-2-methylphenyl)guanidine hydrochloride **15** (0.399 g, 1.64 mmol) in *n*-butanol (10 mL) was added *(E)-3-(dimethylamino)-1-(pyridin-3-yl)prop-2-en-1-one* **10** (0.240 g, 1.36 mmol) and NaOH (0.071 g, 1.77 mmol), and the suspension was refluxed for 16 h. The reaction mixture was then cooled to r.t., diluted with H_2_O and extracted with CH_2_Cl_2_. The combined organic layers were washed with brine, dried over MgSO_4_, and concentrated under reduced pressure. The product was purified by flash column chromatography (silica gel, EtOAc) to give 0.391 g (89%) of the intermediate ester **16**. To a solution of **16** (0.391 g, 1.22 mmol) in ethanol (15 mL) was added 5% aq. NaOH (8.8 mL, 10.98 mmol) and the mixture was stirred at 50 °C for 2.5 h. The reaction mixture was concentrated under reduced pressure, cooled to 0 °C, and neutralized with 1N HCl. The precipitated product was filtered off, washed with H_2_O and CH_3_OH, and dried to give **17**. Yield 86%. ^1^H NMR (DMSO-*d*_6_, 400 MHz): δ 12.83 (brs, 1H, OH), 9.30 (s, 1H), 9.09 (s, 1H), 8.73 (s, 1H), 8.56 (d, *J* = 4.8 Hz, 1H), 8.51 (d, *J* = 7.3 Hz, 1H), 8.31 (s, 1H), 7.65 (d, *J* = 7.7 Hz, 1H), 7.60–7.57 (m, 1H), 7.50 (d, *J* = 4.8 Hz, 1H), 7.38 (d, *J* = 7.8 Hz, 1H), 2.34 (s, 3H, CH_3_) ppm.

#### 3.1.18. *N*-Ferrocenyl-4-methyl-3-((4-(pyridin-3-yl)pyrimidin-2-yl)amino)benzamide (**18**)

A solution of carboxylic acid **17** (0.080 g, 0.250 mmol), DIPEA (0.8 mL, 0.500 mmol), and HATU (0.096 g, 0.250 mmol) in dry DMF (2 mL) was stirred at r.t. for 15 min under an argon atmosphere. Ferroceneamine (0.050 g, 0.250 mmol) was added and the reaction was stirred at r.t. for 3 h. The mixture was diluted with H_2_O and extracted with CH_2_Cl_2_. The combined organic layers were washed with water and brine, dried over MgSO_4_, and concentrated under reduced pressure. The product was purified by flash column chromatography (silica gel, CH_2_Cl_2_/CH_3_OH/NH_4_OH = 30:1:0.1). Yield 91%. Mp > 117 °C decomposition. ^1^H NMR (CDCl_3_, 400 MHz): δ 9.18 (d, *J* = 1.8 Hz, 1H), 8.65 (dd, *J* = 4.8; 1.6 Hz, 1H), 8.61 (d, *J* = 1.6 Hz, 1H), 8.46 (d, *J* = 5.2 Hz, 1H), 8.34 (dt, *J* = 8.3; 1.8 Hz, 1H), 7.43–7.35 (m, 3H), 7.23 (d, *J* = 7.9 Hz, 1H), 7.13 (d, *J* = 5.2 Hz, 1H), 7.05 (brs, 1H, NH), 4.68 (s, 5H, Cp-H), 4.11 (s, 5H, Cp5), 3.98 (t, *J* = 1.7 Hz, 2H, Cp-H), 2.33 (s, 3H, CH_3_) ppm. ^13^C NMR (CDCl_3_, 100.6 MHz): δ 165.51 (C), 162.75 (C), 160.53 (C), 159.16 (CH), 151.63 (CH), 148.43 (CH), 137.74 (C), 134.61 (CH), 133.57 (C), 132.59 (C), 132.05 (C), 130.81 (CH), 123.86 (CH), 121.85 (CH), 119.90 (CH), 108.67 (CH), 95.03 (C), 69.28 (5 CH, Cp5), 64.75 (2 CH, Cp), 61.69 (2 CH, Cp), 18.21 18.09 (CH_3_) ppm. HRMS (HESI): found for C_27_H2_3_FeN_5_O [M + H]^+^ *m*/*z* 490.1321, calcd. *m*/*z* 490.1252.

### 3.2. Cell Lines and Culture Conditions

In vitro cytotoxicity was evaluated using a panel of human BCR–ABL-positive leukemic cell lines (AR-230, BV-173, LAMA-84, and K-562), along with normal fibroblast cells (CCL-1) as a non-malignant control. All cell lines were obtained from the German Collection of Microorganisms and Cell Cultures (DSMZ GmbH, Braunschweig, Germany). Cells were maintained in RPMI 1640 medium supplemented with 10% fetal bovine serum (FBS) and 5% L-glutamine. Cultures were incubated at 37 °C in a humidified atmosphere containing 5% CO_2_ under standard conditions.

### 3.3. Cell Viability Assay

Cytotoxic effects of the newly synthesized ferrocene derivatives were assessed in comparison to imatinib, used as a reference compound. Cell viability was determined using the MTT colorimetric assay. Cells in the exponential growth phase were seeded into 96-well plates (100 μL per well) at densities of 3 × 10^5^ cells for suspension lines and 1.5 × 10^5^ for the adherent fibroblast line (CCL-1). Treatments involved exposure to a range of compound concentrations (0.5–200 μM) for 72 h. Following incubation, 5 mg/mL MTT solution (prepared in PBS and filter sterilized) was added to each well. After an additional 1–4 h, during which purple formazan crystals formed, the precipitate was solubilized in isopropanol containing 5% formic acid. Absorbance was measured at 550 nm, with background correction using MTT and isopropanol blanks. Data were normalized to the mean absorbance of untreated controls, set as 100% viability. Semi-logarithmic dose–response curves were generated, and IC_50_ values were calculated for each compound and cell line. Statistical significance was determined at *p* ≤ 0.05.

### 3.4. Docking Analysis

The ferrocene-containing analogs to imatinib and nilotinib (**6**, **9**, **14**, and **18**) (Figure 2) were modeled using a ferrocene moiety derived from the crystallographic structure of a homobiotin derivative (PDB ID: 5MYQ using Avogadro 1.2.0 [26]. Using chain B of the 2HYY [23] crystal structure of imatinib complexed with c-Abl tyrosine kinase (TK), the missing E275 residue was added using MODELLER9.25 [27]. Ligands were docked into the ATP binding site of the kinase using the 2HYY reference ligand to define the binding site. Docking was performed with Gnina 1.0 [24] using default settings—8 Monte Carlo chains were run during sampling, adding 4 Å of buffer space to the auto-generated box defined by the reference ligand, and expanding the box by an additional 1 Å when necessary to allow the input ligand conformation to freely rotate within the box. The reference imatinib structure was redocked into its cognate protein conformation using an identical protocol. It was found that imatinib binds within the ATP binding site in its protonated state at the nitrogen atom of the NMe group in the 4-methyl-piperazine moiety [28,29]. Therefore, the monoprotonated form of imatinib was used for docking calculations. Each predicted pose generated via Gnina provides two estimated affinities: the affinity, expressed in kcal/mol, and the CNN affinity, a convolutional neural network estimate of the affinity, expressed in pK units.

## 4. Conclusions

This study highlights the promise of Fc-substituted analogues of imatinib and nilotinib as novel BCR-ABL1 inhibitors with potential advantages in overcoming drug resistance and enhancing selectivity. While Fc incorporation can disrupt canonical binding interactions, it also introduces beneficial redox properties and alternative binding modes that preserve or even enhance biological activity in specific leukemia subtypes. The strong correlation between experimental and computational LEs reinforces the predictive value of docking-based affinity models. Collectively, these results validate Fc as a versatile scaffold for rational drug design in targeted leukemia therapy.

## Figures and Tables

**Table 1:** IC_50_ values (µM) ± standard deviations of the novel ferrocene derivatives of imatinib and nilotinib. The anticancer activities of these compounds, along with the positive control, imatinib, were evaluated against four leukemia cell lines (BV-173, K-562, AR-230, and LAMA-84), and their corresponding ligand efficiency (LE) values are presented.

Compound	BV-173	LE_BV173_	K-562	LE_K562_	AR-230	LE_AR230_	LAMA-84	LE_LAMA84_	CCL-1
**6**	33.6 ± 2.1	0.107	29.9 ± 3.1	0.102	5.9 ± 0.2	0.125	25.4 ± 2.9	0.109	55.7 ± 9.5
**9**	33.6 ± 2.3	0.102	25.9 ± 2.9	0.104	143.8 ± 10.6	0.087	25.4 ± 2.4	0.104	>200
**14**	17.9 ± 3.1	0.140	>200		5.1 ± 1.3	0.156	27.3 ± 1.8	0.134	>200
**18**	15.1 ± 2.9	0.142	>200		5.1 ± 0.9	0.156	26.8 ± 3.2	0.134	60.2 ± 8.3
imatinib	22.8 ± 4.8	0.125	45.5 ± 5.0	0.117	4.7 ± 1.0	0.144	0.5 ± 0.05	0.170	>200

**Table 2:** The predicted affinities and CNN affinities, of the novel ferrocene derivatives and the reference compound, imatinib, resulting from molecular docking are presented, along with the corresponding LE_AFF_ and LE_CNN_.

Compound	Affinity, kcal/mol	CNN Affinity, pK Units	LE_AFF_	LE_CNN_
**6**	−8.35	7.552	0.200	0.180
**9**	−8.39	6.946	0.191	0.158
**14**	−10.17	7.337	0.299	0.216
**18**	−8.06	7.023	0.237	0.207
imatinib	−13.14	7.864	0.355	0.213

**Table 3:** The correlations between the experimental (LE_BV173_, LE_K562_, LE_AR230_, and LE_LAMA84_) and the predicted (LE_AFF_ and LE_CNN_) LEs are presented.

	LE_BV173_	LE_K562_	LE_AR230_	LE_LAMA84_
LE_AFF_	0.569	0.977	0.635	0.948
LE_CNN_	0.896	0.989	0.961	0.809

## Data Availability

Data are contained within the article and Supplementary Materials.

## Supplementary Materials

The following supporting information can be downloaded at https://www.mdpi.com/article/10.3390/molecules30153101/s1: Copies of 1H and 13C NMR spectra, and HRMS data of the targeted compounds.
