# Supplementary material for: Design, Docking Analysis, and Structure–Activity Relationship of Ferrocene-Modified Tyrosine Kinase Inhibitors: Insights into BCR-ABL Interactions
Source: Molecules. 2025 Jul 24;30(15):3101. doi: 10.3390/molecules30153101 (PMC12348835; doi:10.3390/molecules30153101)
Supplement: Supplementary file 1 [file molecules-30-03101-s001.zip › molecules-3755855-supplementary.pdf]

Supporting Information

Targeting BCR-ABL1 with Ferrocene-Modified Tyrosine Kinase Inhibitors: Structure–Activity Insights

Irena Philipova [1], Maryana Atanasova [2], Rositsa Mihaylova [2], Asine Dailova-Barzeva [2], Stefan Ivanov [2], Rumyana Simeonova [2]* and Georgi Stavrakov [1,2]*

[1] Institute of Organic Chemistry with Centre of Phytochemistry, Bulgarian Academy of Sciences, Acad. G. Bontchev str. Bl. 9, 1113 Sofia, Bulgaria; irena.philipova@orgchm.bas.bg

[2] Faculty of Pharmacy, Medical University of Sofia, 1000 Sofia, Bulgaria; matanasova@pharmfac.mu-sofia.bg; rmihaylova@pharmfac.mu-sofia.bg; asinedailova61@gmail.com; sivanov@ddg-pharmfac.net; rsimeonova@pharmfac.mu-sofia.bg; stavrakov@pharmfac.mu-sofia.bg

* Correspondence: rsimeonova@pharmfac.mu-sofia.bg; stavrakov@pharmfac.mu-sofia.bg

Copies of ^{1}H and ^{13}C NMR spectra, and HRMS data of the target compounds.

*N-(4-methyl-3-((4-ferrocenylpyrimidin-2-yl)amino)phenyl)-4-((4-methylpiperazin-1-yl)methyl)benzamide (**6**)*

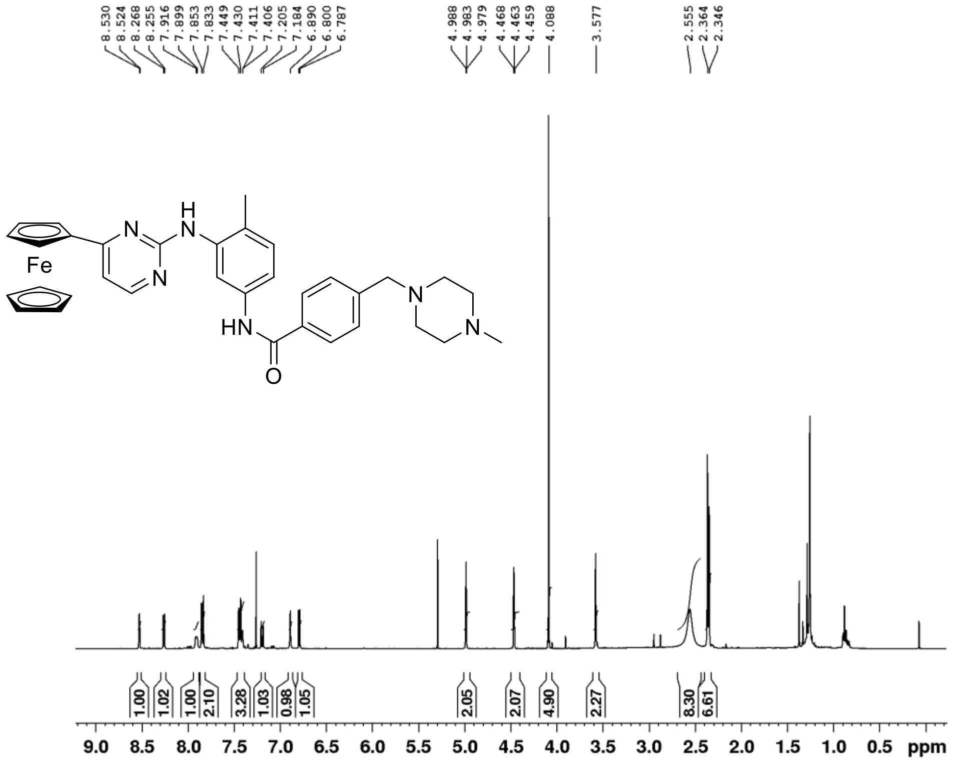

Current Data Parameters
NAME FM01802
EXPNO 11
PROCNO 1

F2 - Acquisition Parameters
Date_ 20230208
Time 10.27 h
INSTRUM Avance Neo 400
PROBHD Z175272_0007 (
PULPROG zg30
TD 32768
SOLVENT CDCl3
NS 32
DS 0
SWH 6578.947 Hz
FIDRES 0.401547 Hz
AQ 2.4903679 sec
RG 101
DW 76.000 usec
DE 7.79 usec
TE 298.0 K
D1 2.00000000 sec
TD0 1
SFO1 400.1330415 MHz
NUC1 1H
P0 3.33 usec
P1 10.00 usec
PLW1 20.07200050 W

F2 - Processing parameters
SI 65536
SF 400.1300098 MHz
WDW EM
SSB 0
LB 0 Hz
GB 0
PC 1.00

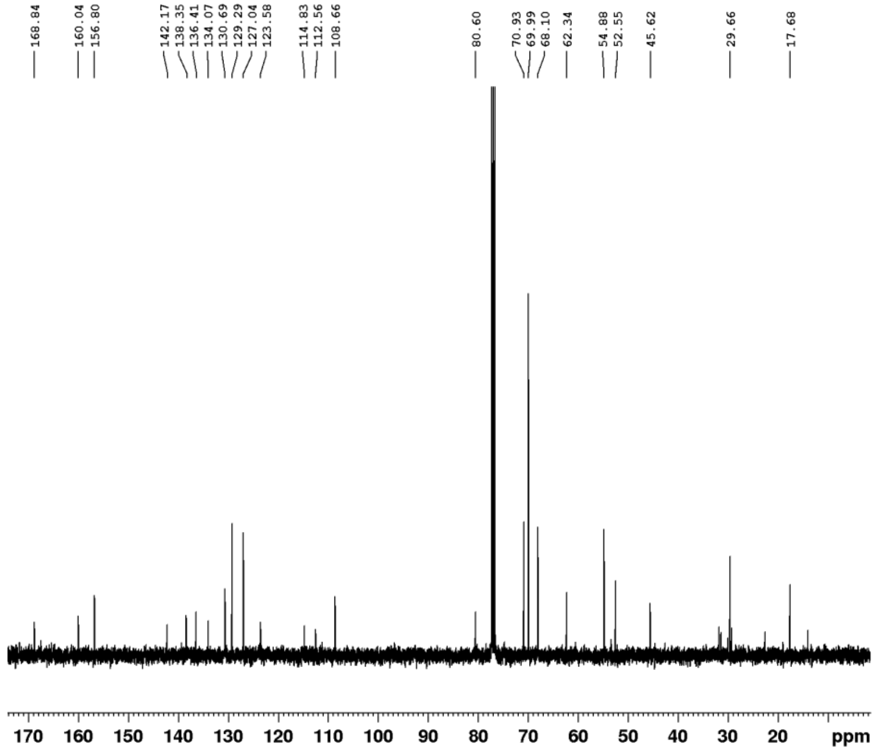

Current Data Parameters
NAME FM01802
EXPNO 12
PROCNO 1

F2 - Acquisition Parameters
Date_ 20230208
Time 10.37 h
INSTRUM Avance Neo 400
PROBHD Z175272_0007 (
PULPROG zgdc30
TD 32768
SOLVENT CDCl3
NS 256
DS 0
SWH 23809.524 Hz
FIDRES 1.453218 Hz
AQ 0.6881280 sec
RG 22.6
DW 21.000 usec
DE 6.50 usec
TE 298.0 K
D1 1.50000000 sec
D11 0.03000000 sec
TD0 1
SFO1 100.6238383 MHz
NUC1 13C
P0 3.33 usec
P1 10.00 usec
PLW1 58.46900177 W
SFO2 400.1316008 MHz
NUC2 1H
CPDPRG[2 waltz16
PCPD2 90.00 usec
PLW2 20.07200050 W
PLW12 0.24781001 W

F2 - Processing parameters
SI 65536
SF 100.6127730 MHz
WDW EM
SSB 0
LB 1.00 Hz
GB 0
PC 1.40

HRMS data of compound **6**.

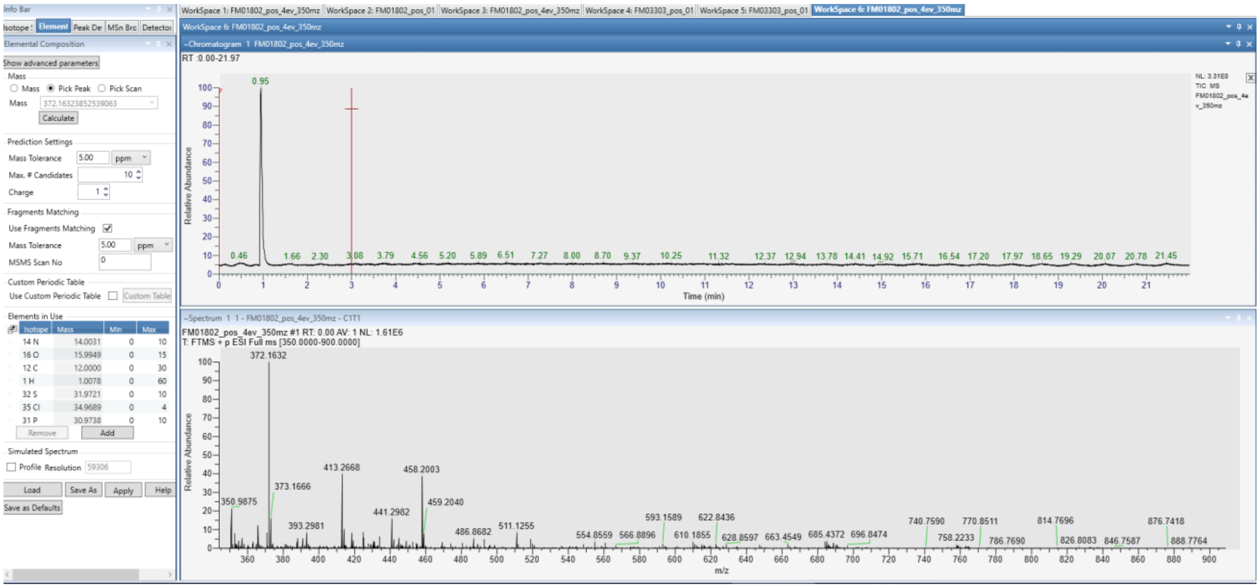

D:\DATA\...\FM01802_pos_4ev_350mz 07/27/23 13:45:06

FM01802_pos_4ev_350mz#214-219 RT: 0.94-0.96 AV: 6
T: FTMS + p ESI Full ms [350.0000-900.0000]

m/z	Intensity	Relative
416.88105	2098307.5	7.15
430.86188	6490710.5	22.10
436.86606	9242858.0	31.48
445.11997	2957878.3	10.07
452.84369	3107912.3	10.58
458.84789	4632427.5	15.78
519.13879	2189882.5	7.46
534.83057	2824404.3	9.62
550.80660	2337722.3	7.96
601.23733	29363768.0	100.00
602.23990	11880921.0	40.46
603.24307	2243904.5	7.64
647.45873	3450825.3	11.75
764.57420	3177979.3	10.82

4-methyl-N-(3-(4-methyl-1H-imidazol-1-yl)-5-(trifluoromethyl)phenyl)-3-((4-ferrocenylpyrimidin-2-yl)amino)benzamide ***(9)***

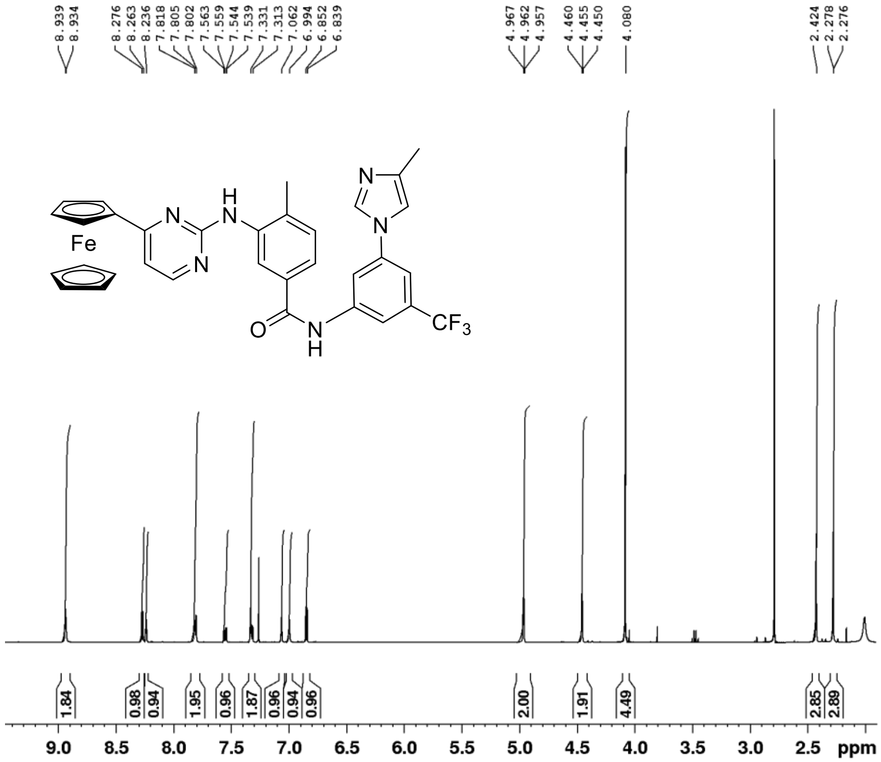


```
Current Data Parameters
NAME              FM03405
EXPNO                  11
PROCNO                  1

F2 - Acquisition Parameters
Date_            20230613
Time                10.29 h
INSTRUM    Avance Neo 400
PROBHD     Z175272_0007 (
PULPROG              zg30
TD                  32768
SOLVENT             CDCl3
NS                     32
DS                      0
SWH              6578.947 Hz
FIDRES           0.401547 Hz
AQ              2.4903679 sec
RG                    101
DW                 76.000 usec
DE                   7.79 usec
TE                  298.1 K
D1             2.00000000 sec
TD0                     1
SFO1          400.1330415 MHz
NUC1                   1H
P0                   3.33 usec
P1                  10.00 usec
PLW1          20.07200050 W

F2 - Processing parameters
SI                  65536
SF            400.1300096 MHz
WDW                    no
SSB                     0
LB                      0 Hz
GB                      0
PC                   1.00
```

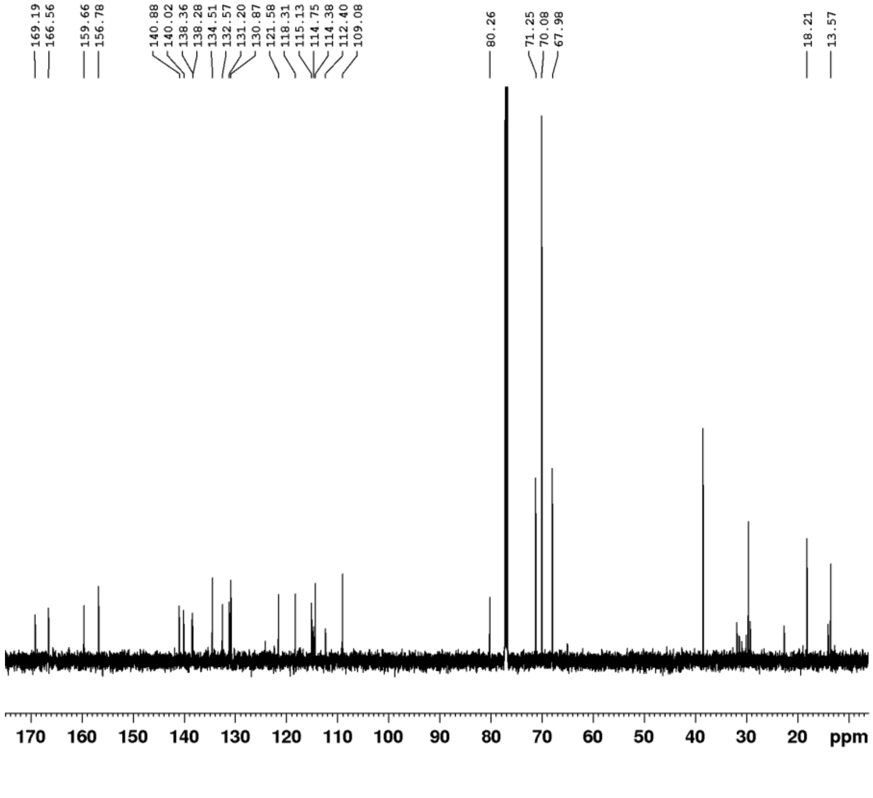


```
Current Data Parameters
NAME              FM03303
EXPNO                  12
PROCNO                  1

F2 - Acquisition Parameters
Date_            20230522
Time                16.16 h
INSTRUM            Avance
PROBHD     Z168773_0033 (
PULPROG            zgdc30
TD                  32768
SOLVENT             CDCl3
NS                    256
DS                      0
SWH             35714.286 Hz
FIDRES           2.179827 Hz
AQ              0.4587520 sec
RG                    101
DW                 14.000 usec
DE                  18.00 usec
TE                  298.0 K
D1             1.50000000 sec
D11            0.03000000 sec
TD0                     1
SFO1          150.9319844 MHz
NUC1                  13C
P0                   3.33 usec
P1                  10.00 usec
PLW1          67.54000092 W
SFO2          600.1824007 MHz
NUC2                   1H
CPDPRG[2          waltz16
PCPD2               80.00 usec
PLW2          21.72701073 W
PLW12          0.49773711 W

F2 - Processing parameters
SI                  65536
SF            150.9153879 MHz
WDW                    EM
SSB                     0
LB                   1.00 Hz
GB                      0
PC                   1.40
```

HRMS data of compound **9**.

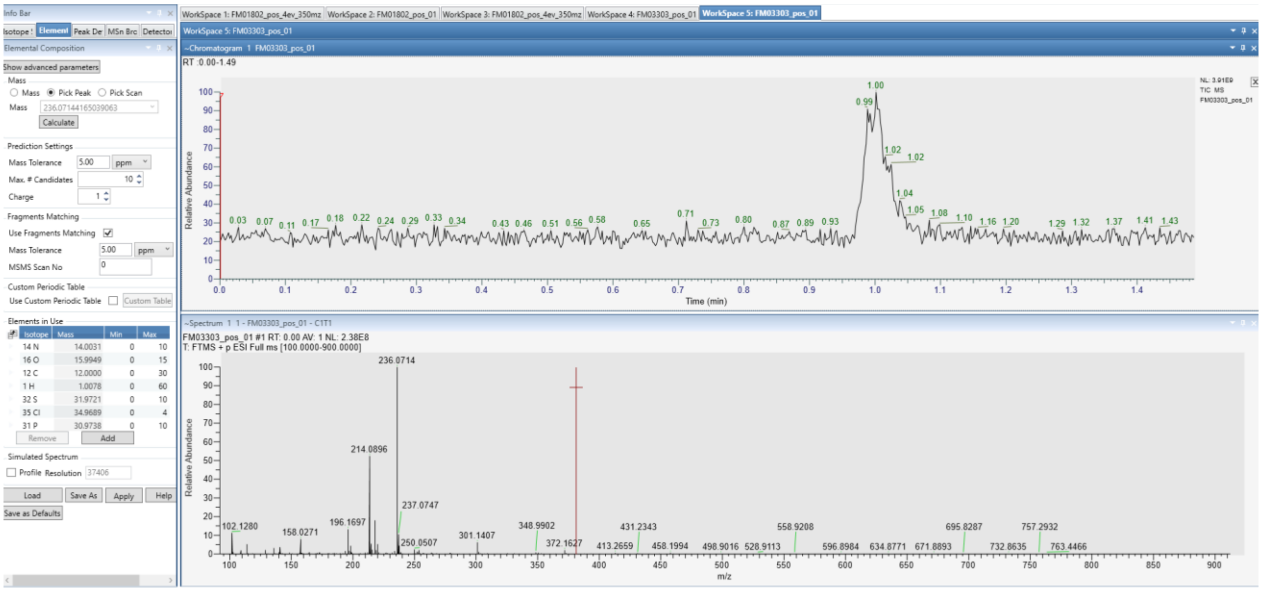

D:\DATA\...\IrenaFilipova\FM03303_pos_01 07/27/23 13:06:08

FM03303_pos_01#436-455 RT: 0.98-1.02 AV: 20
SB: 48 0.84-0.94
T: FTMS + p ESI Full ms [100.0000-900.0000]

m/z	Intensity	Relative
102.12801	636713984.0	100.00
103.13129	46717636.0	7.34
104.10715	245957136.0	38.63
105.11048	14371517.0	2.26
117.10236	5805788.0	0.91
118.08642	42666804.0	6.70
142.94808	6470701.5	1.02
196.16941	5523318.5	0.87
200.98343	8066169.0	1.27
318.08689	40232148.0	6.32
318.58940	13388914.0	2.10
319.08439	630334912.0	99.00
319.58552	253297024.0	39.78
320.08700	50669060.0	7.96
320.58820	6984876.0	1.10
353.26578	8646875.0	1.36
381.29721	7395864.5	1.16
516.62087	7242229.5	1.14
637.16200	33047756.0	5.19
638.16475	12942189.0	2.03

N-(4-methyl-3-((4-(pyridin-3-yl)pyrimidin-2-yl)amino)phenyl)ferrocene carboxamide ***(14)***

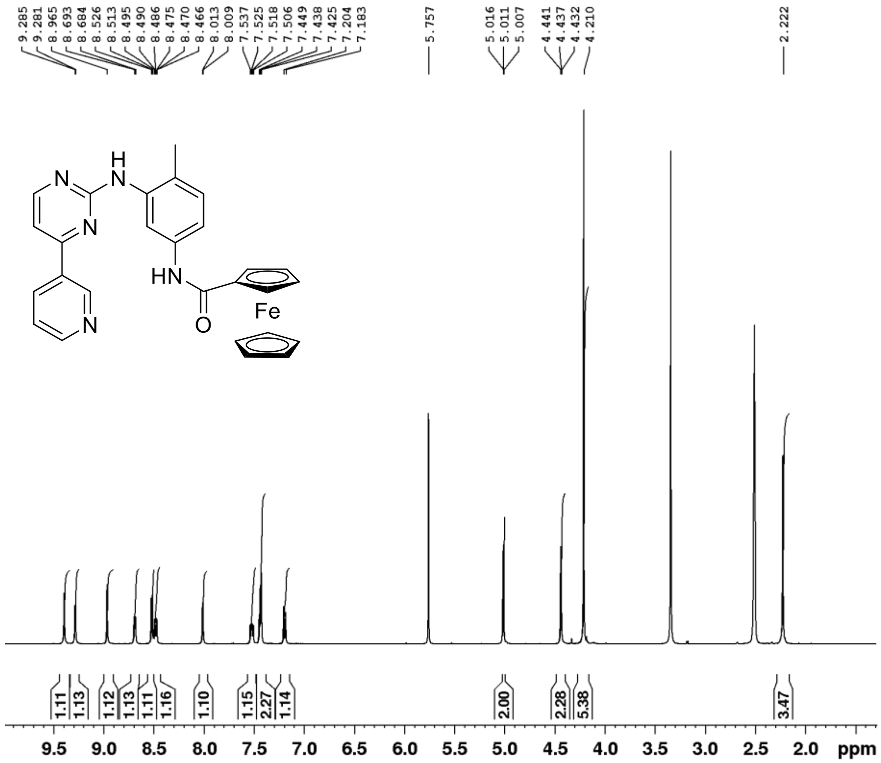

Current Data Parameters
NAME GC1712
EXPNO 11
PROCNO 1

F2 - Acquisition Parameters
Date_ 20220407
Time 17.02 h
INSTRUM Avance Neo 400
PROBHD Z175272_0007 (
PULPROG zg30
TD 32768
SOLVENT DMSO
NS 32
DS 0
SWH 6578.947 Hz
FIDRES 0.401547 Hz
AQ 2.4903679 sec
RG 101
DW 76.000 usec
DE 7.79 usec
TE 298.0 K
D1 2.00000000 sec
TD0 1
SFO1 400.1330415 MHz
NUC1 1H
P0 3.33 usec
P1 10.00 usec
PLW1 20.07200050 W

F2 - Processing parameters
SI 65536
SF 400.1300000 MHz
WDW EM
SSB 0
LB 0 Hz
GB 0
PC 1.00

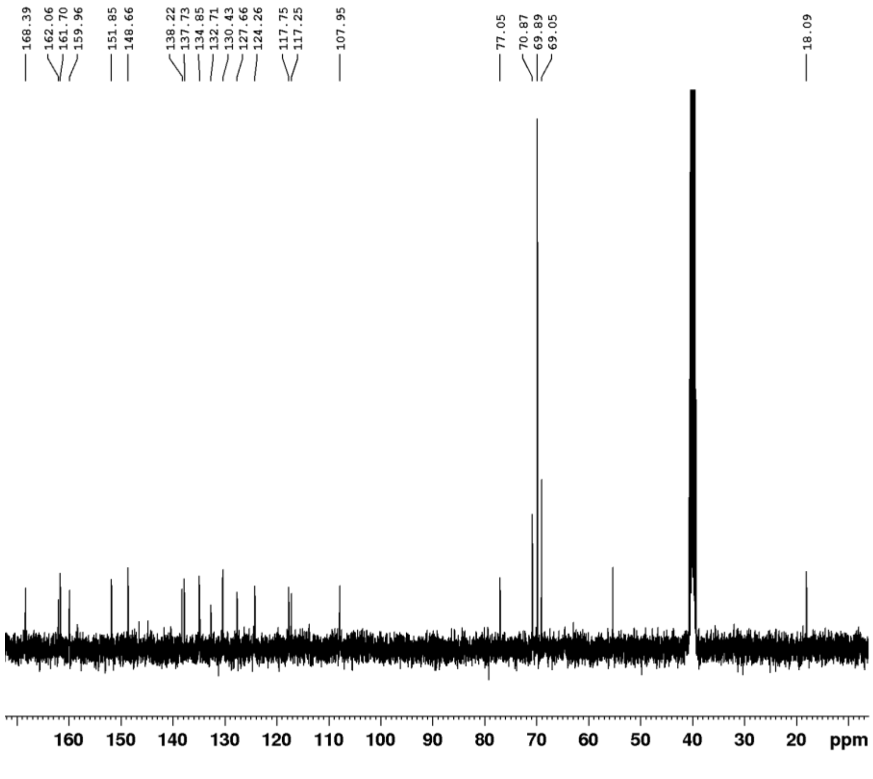

Current Data Parameters
NAME GC1712
EXPNO 12
PROCNO 1

F2 - Acquisition Parameters
Date_ 20220407
Time 17.07 h
INSTRUM Avance Neo 400
PROBHD Z175272_0007 (
PULPROG zgdc30
TD 32768
SOLVENT DMSO
NS 128
DS 0
SWH 23809.523 Hz
FIDRES 1.453218 Hz
AQ 0.6881280 sec
RG 22.6
DW 21.000 usec
DE 6.50 usec
TE 298.0 K
D1 1.50000000 sec
D11 0.03000000 sec
TD0 1
SFO1 100.6238383 MHz
NUC1 13C
P0 3.33 usec
P1 10.00 usec
PLW1 58.46900177 W
SFO2 400.1316008 MHz
NUC2 1H
CPDPRG[2 waltz16
PCPD2 90.00 usec
PLW2 20.07200050 W
PLW12 0.24781001 W

F2 - Processing parameters
SI 65536
SF 100.6127690 MHz
WDW EM
SSB 0
LB 1.00 Hz
GB 0
PC 1.40

HRMS data of compound **14**.

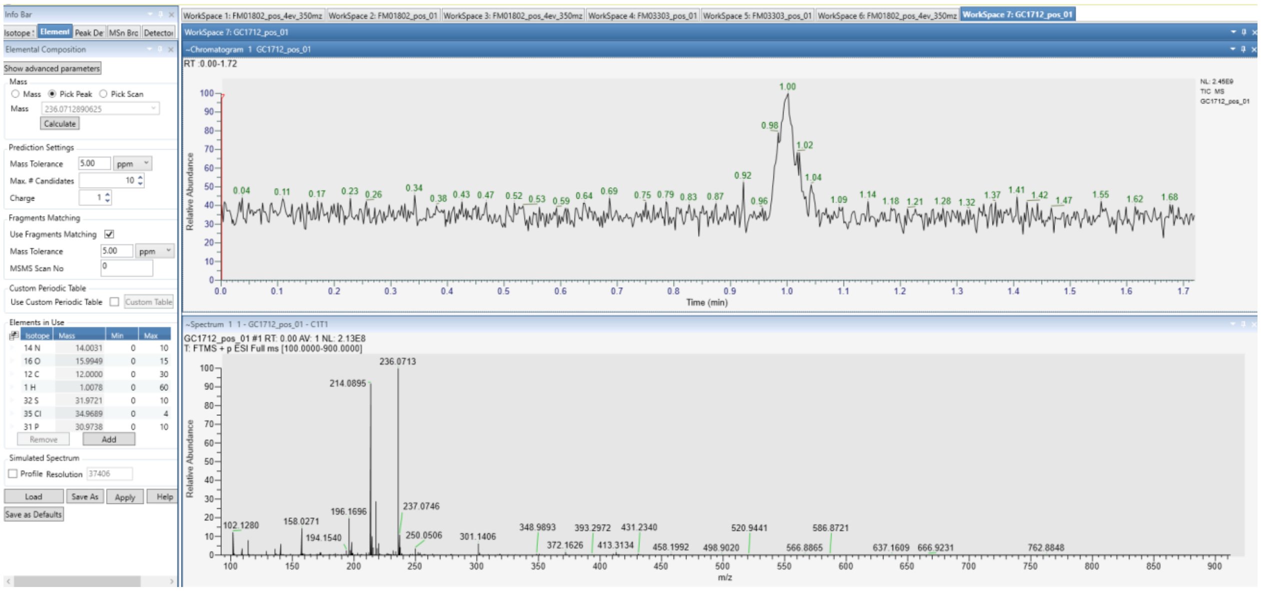

D:\DATA\...\IrenaFilipova\GC1712_pos_01 07/27/23 13:08:04

GC1712_pos_01#438-450 RT: 0.99-1.01 AV: 13
SB: 29 0.83-0.90
T: FTMS + p ESI Full ms [100.0000-900.0000]

m/z	Intensity	Relative
212.99891	3101842.0	0.71
262.90660	4096901.3	0.93
278.13979	3046275.5	0.69
353.26561	8675743.0	1.98
381.29713	7229543.0	1.65
488.13683	22133440.0	5.04
489.14255	6128169.5	1.40
490.13192	353346752.0	80.50
491.13493	116529624.0	26.55
492.13760	19548890.0	4.45
493.14005	2402226.3	0.55
512.11368	23661984.0	5.39
513.11667	8016160.0	1.83

N-Ferrocenyl-4-methyl-3-((4-(pyridin-3-yl)pyrimidin-2-yl)amino)benzamide ***(18)***

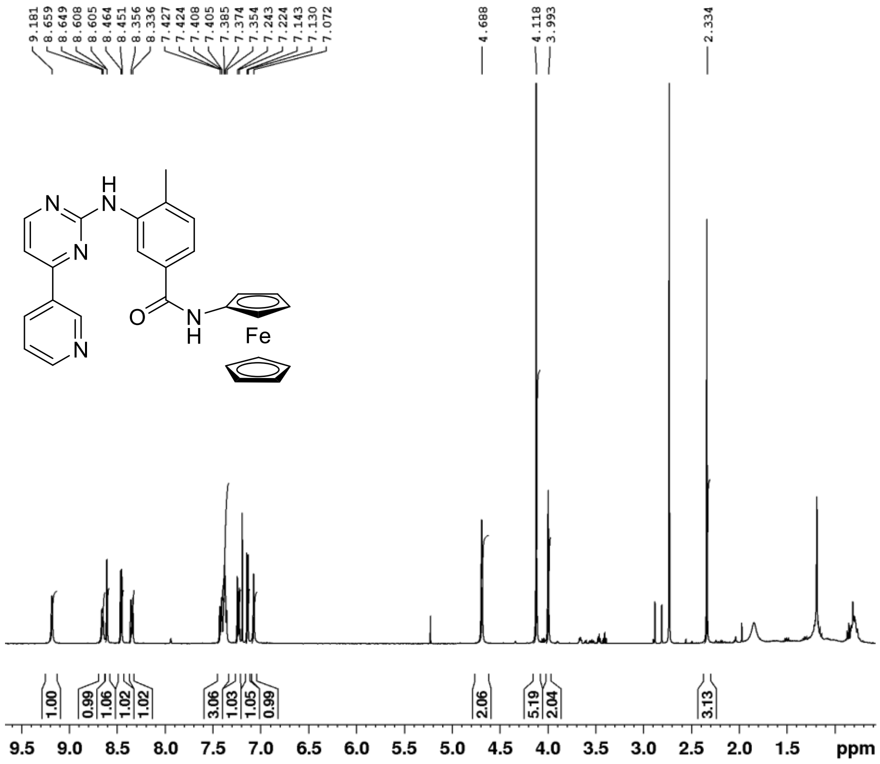

**Current Data Parameters**	
NAME	FM02802
EXPNO	11
PROCNO	1
**F2 - Acquisition Parameters**	
Date_	20230404
Time	17.10 h
INSTRUM	Avance Neo 400
PROBHD	Z175272_0007 (
PULPROG	zg30
TD	32768
SOLVENT	CDCl3
NS	32
DS	0
SWH	6578.947 Hz
FIDRES	0.401547 Hz
AQ	2.4903679 sec
RG	101
DW	76.000 usec
DE	7.79 usec
TE	298.0 K
D1	2.00000000 sec
TD0	1
SFO1	400.1330415 MHz
NUC1	1H
P0	3.33 usec
P1	10.00 usec
PLW1	20.07200050 W
**F2 - Processing parameters**	
SI	65536
SF	400.1300369 MHz
WDW	EM
SSB	0
LB	0 Hz
GB	0
PC	1.00

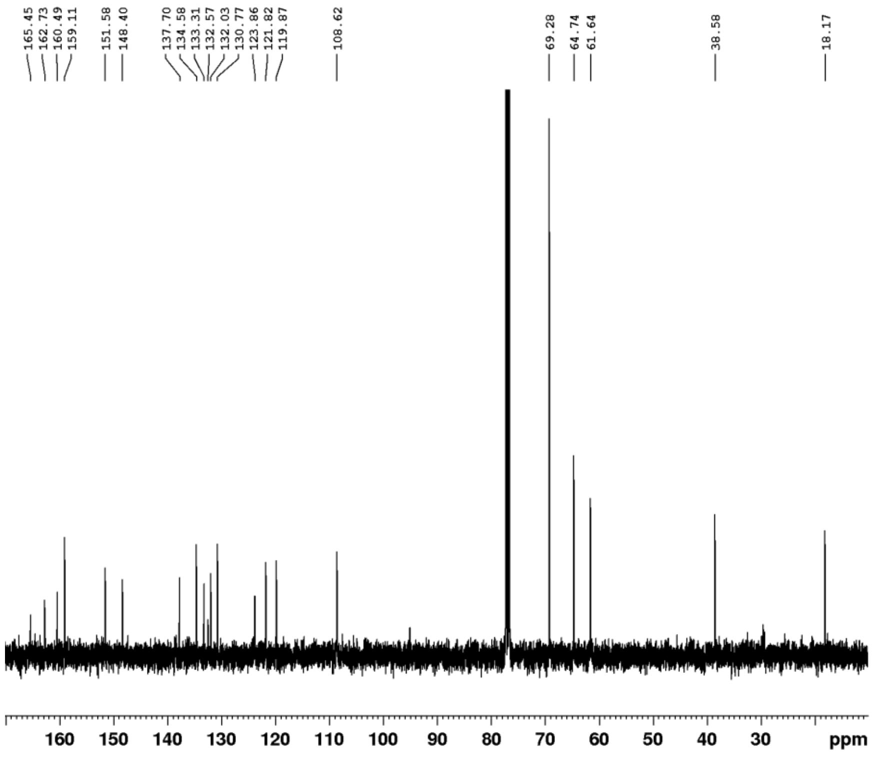

**Current Data Parameters**	
NAME	FM02802
EXPNO	12
PROCNO	1
**F2 - Acquisition Parameters**	
Date_	20230404
Time	17.20 h
INSTRUM	Avance Neo 400
PROBHD	Z175272_0007 (
PULPROG	zgdc30
TD	32768
SOLVENT	CDCl3
NS	256
DS	0
SWH	23809.524 Hz
FIDRES	1.453218 Hz
AQ	0.6881280 sec
RG	22.6
DW	21.000 usec
DE	6.50 usec
TE	298.0 K
D1	1.50000000 sec
D11	0.03000000 sec
TD0	1
SFO1	100.6238383 MHz
NUC1	13C
P0	3.33 usec
P1	10.00 usec
PLW1	58.46900177 W
SFO2	400.1316008 MHz
NUC2	1H
CPDPRG[2	waltz16
PCPD2	90.00 usec
PLW2	20.07200050 W
PLW12	0.24781001 W
**F2 - Processing parameters**	
SI	65536
SF	100.6127730 MHz
WDW	EM
SSB	0
LB	1.00 Hz
GB	0
PC	1.40

HRMS data of compound **18**.

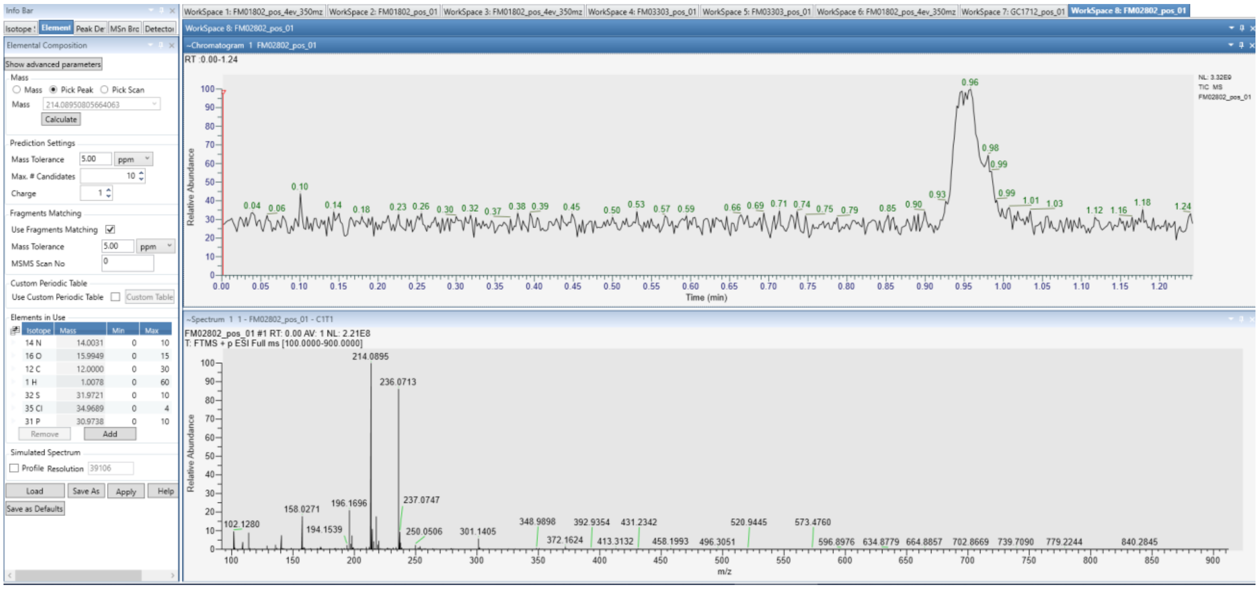

D:\DATA\...\IrenaFilipova\FM02802_pos_01 07/27/23 13:04:25

FM02802_pos_01#416-433 RT: 0.93-0.97 AV: 18
T: FTMS + p ESI Full ms [100.0000-900.0000]

m/z	Intensity	Relative
158.02706	28261078.0	3.53
196.16951	44782716.0	5.59
198.18511	16382342.0	2.04
200.98341	11991440.0	1.50
214.08945	189650480.0	23.67
215.09295	20055562.0	2.50
236.07129	81859328.0	10.22
424.08432	33193916.0	4.14
488.13724	23636194.0	2.95
489.12260	16691698.0	2.08
490.13215	378483936.0	47.24
491.13530	124074624.0	15.48
492.13805	20847414.0	2.60
512.11407	12251475.0	1.53
